# α-SNAP Prevents Docking of the Acrosome during Sperm Exocytosis because It Sequesters Monomeric Syntaxin

**DOI:** 10.1371/journal.pone.0021925

**Published:** 2011-07-18

**Authors:** Facundo Rodríguez, Matías A. Bustos, María N. Zanetti, María C. Ruete, Luis S. Mayorga, Claudia N. Tomes

**Affiliations:** Laboratorio de Biología Celular y Molecular, Instituto de Histología y Embriología, Facultad de Ciencias Médicas, Universidad Nacional de Cuyo, Mendoza, Argentina; University of Geveva, Switzerland

## Abstract

α-SNAP has an essential role in membrane fusion that consists of bridging *cis* SNARE complexes to NSF. α-SNAP stimulates NSF, which releases itself, α-SNAP, and individual SNAREs that subsequently re-engage in the *trans* arrays indispensable for fusion. α-SNAP also binds monomeric syntaxin and NSF disengages the α-SNAP/syntaxin dimer. Here, we examine why recombinant α-SNAP blocks secretion in permeabilized human sperm despite the fact that the endogenous protein is essential for membrane fusion. The only mammalian organism with a genetically modified α-SNAP is the *hyh* mouse strain, which bears a M105I point mutation; males are subfertile due to defective sperm exocytosis. We report here that recombinant α-SNAP-M105I has greater affinity for the cytosolic portion of immunoprecipitated syntaxin than the wild type protein and in consequence NSF is less efficient in releasing the mutant. α-SNAP-M105I is a more potent sperm exocytosis blocker than the wild type and requires higher concentrations of NSF to rescue its effect. Unlike other fusion scenarios where SNAREs are subjected to an assembly/disassembly cycle, the fusion machinery in sperm is tuned so that SNAREs progress uni-directionally from a *cis* configuration in resting cells to monomeric and subsequently *trans* arrays in cells challenged with exocytosis inducers. By means of functional and indirect immunofluorescense assays, we show that recombinant α-SNAPs — wild type and M105I — inhibit exocytosis because they bind monomeric syntaxin and prevent this SNARE from assembling with its cognates in *trans*. Sequestration of free syntaxin impedes docking of the acrosome to the plasma membrane assessed by transmission electron microscopy. The N-terminal deletion mutant α-SNAP-(160–295), unable to bind syntaxin, affects neither docking nor secretion. The implications of this study are twofold: our findings explain the fertility defect of *hyh* mice and indicate that assembly of SNAREs in *trans* complexes is essential for docking.

## Introduction

Regulated exocytosis of sperm's single dense-core secretory vesicle (termed the acrosome reaction, AR) relies on highly conserved molecules that drive intracellular membrane fusion and exocytosis in most cells. All fusion reactions are driven by supramolecular protein complexes that transiently and cyclically assemble and disassemble. At the core of this machinery we find the Rab and SNARE families as well as a number of proteins that interact with SNAREs, such as N-ethylmaleimide-sensitive factor (NSF), soluble NSF-attachment proteins (SNAPs), Munc18, complexins, and synaptotagmins [1]–[7]. SNAREs are integral membrane proteins characterized by sequences (the SNARE motifs) that are unstructured when monomeric. These motifs exhibit a high propensity to form coiled coil structures of extraordinary stability when appropriate sets of SNAREs interact with each other. SNARE complexes consist of four intertwined, parallel helices, each supplied by a different SNARE motif. According to the most widespread current model, membrane fusion is driven by the energy released during the formation of four helix bundles between SNARE proteins residing in the two membranes that are undergoing fusion (*trans* SNARE complexes) [8]. When all cognate SNAREs are located in the same membrane, they spontaneously assemble in stable *cis* complexes, which are functionally inactive. Disentangling these complexes to regenerate monomeric SNAREs that will subsequently be available to engage in productive *trans* complexes requires considerable metabolic energy; this energy is provided by NSF via the hydrolysis of ATP. NSF does not bind directly to SNARE complexes but requires the assistance of cofactors that bridge the two: the SNAP proteins, which, when part of these bridges, stimulate the ATPase activity of NSF [8], [9]. It is believed that the activity of NSF is constitutive in most cells to ensure that *cis* complexes are constantly disassembled under normal steady-state conditions to regenerate free SNAREs in membranes. It has recently been shown that SNAPs support synaptic vesicle priming in a subset of hippocampal neurons by determining the availability of free SNAREs [10]. In sperm, however, the picture is somewhat different, perhaps owing to their need to coordinate the AR carefully with the exact moment when they encounter the egg. Thus, under resting conditions SNAREs do not cycle but are engaged in *cis* complexes on both plasma and outer acrosomal membranes [11], as NSF's dissociating activity is repressed by tyrosine phosphorylation [12]. Upon sperm activation, extracellular calcium enters the cytoplasm and indirectly activates Rab3A [13], [14]. Subsequently, protein tyrosine phosphatase 1B (PTP1B) dephosphorylates NSF, which binds α-SNAP and disassembles *cis* SNARE complexes. Free SNAREs are now able to re-assemble in *trans*, a process that is facilitated by complexin [15], [16]. The acrosome behaves as an internal store of releasable calcium [17]–[20]; efflux from this reservoir through inositol 1,4,5-trisphosphate (IP_3_)-sensitive channels is required for the AR initiated by calcium itself, Rab3A-guanosine 5′-3-*O*-(thio) triphosphate (GTP-γ-S) and cAMP [11], [17], [21], [22]. This local increase in calcium activates synaptotagmin, which displaces complexin from *trans* SNARE complexes and triggers the final steps of membrane fusion [15].

Although the best known receptors for α-SNAP are *cis* SNARE complexes on native or artificial membranes and heterotrimers assembled *in vitro* from purified syntaxin, SNAP-25, and synaptobrevin, several laboratories have demonstrated that recombinant (free) syntaxin also binds α-SNAP *in vitro*, albeit with lower affinity than for SNARE complexes [23]–[27]. Colocalization analysis in PC12 cell membranes indicates that native syntaxin also serves as an α-SNAP receptor [28]. As is the case with *cis* SNARE complexes, NSF releases α-SNAP from both recombinant and native syntaxin [23], [24], [27].

α-SNAP is essential for sperm exocytosis [21], [29]. Nevertheless, addition of relatively large quantities of recombinant α-SNAP to permeabilized human sperm inhibits calcium-triggered secretion; NSF reverses this effect [29]. We know of two other systems where exogenous α-SNAP blocks fusion in native membranes. In yeast, recombinant Sec17p (the yeast homologue of α-SNAP) binds to and stabilizes *cis* SNARE complexes on vacuolar membranes, preventing *trans* pairing with SNAREs from a neighbouring vacuole. Addition of Sec18p (the yeast homologue of NSF) releases Sec17p, disassembles *cis* complexes, and restores fusion [30]. Membrane sheets prepared from PC12 cells lack *cis* SNARE complexes [31]. In this model system, exogenous α-SNAP binds to and prevents monomeric syntaxin from entering *trans* SNARE complexes, thus blocking exocytosis; recombinant NSF rescues this effect [28].

A missense substitution of a methionine for isoleucine at position 105 in α-SNAP has been detected in mice affected with hydrocephalus with hop gait (*hyh*) [32], [33]. *Hyh* males exhibit reduced fertility and their sperm have an exocytotic defect despite the fact that the amount and localization of α-SNAP is undistinguishable from that in wild type animals. Wild type α-SNAP — but not α-SNAP-M105I — rescues exocytosis in permeabilized sperm from *hyh* males, suggesting that the mutant is somehow incapable of accomplishing the AR [34]. These findings are puzzling because the NSF-catalyzed SNARE complex disassembly properties of this mutant *in vitro* are similar to those of the wild type protein [32].

In many exocytotic cells, a number of vesicles can be found at very short distances from the plasma membrane; it is believed that these vesicles are the first to exocytose upon stimulation, and therefore constitute the readily releasable pool [35]. No such pool exists in human sperm because they contain a single granule that, under resting conditions, is evenly separated from the plasma membrane in all cells. Because of its size and shape, the acrosome cannot travel in the cytoplasm, as do secretory vesicles in other cells. How then does it contact the plasma membrane to exocytose its contents? At an early stage during exocytosis, the acrosomal contents swell and the acrosomal membrane stretches toward the cell membrane. Swelling leads to the apposition of the outer acrosomal membrane with the adjacent plasma membrane in multiple points; exocytosis culminates with the opening of fusion pores between both membranes at the points of apposition. In most cells, the fusion pore widens, the membrane surrounding the secretory vesicle incorporates into the plasma membrane, and the granule contents discharge. In sperm, however, the pores widen, but since the outer acrosomal membrane is as large as the area of plasma membrane it is fusing with, the result of the pore widening is the fenestration of the fusion membranes and the joining of pores to produce hybrid plasma membrane-outer acrosomal membrane vesicles and tubules to be shed upon completion of exocytosis [36], [37]. We have developed a strategy to halt exocytosis at a stage when sperm exhibit swollen acrosomes with numerous tight appositions (distances between 0 and 8 nm) between the outer acrosomal and plasma membranes [38]. These distances are comparable to those reported in the literature for morphologically docked secretory vesicles [39], [40] and are a hallmark of docked acrosomes.

Here, we describe the biochemical characterization of the syntaxin-binding properties of α-SNAP-M105I, a mutation that confers the subfertile phenotype on *hyh* male mice. We also report that exogenous wild type α-SNAP and α-SNAP-M105I bind sperm's monomeric syntaxin, precluding its interaction with cognate SNAREs. Without *trans* SNARE complexes the acrosome cannot dock to the plasma membrane and therefore exocytosis is halted until NSF releases α-SNAPs from syntaxin, resuming secretion.

## Materials and Methods

### Ethics Statement

We are cognizant of the Argentinean (ANMAT 5330/97) and international (Declaration of Helsinki) principles and bioethical codes, and guarantee that all procedures carried out in conducting the research reported here were in compliance with both. Human subjects were involved in this project for the purpose of semen donation. The subject population consisted of healthy male donors 21 years of age or over. All donors signed a written Informed Consent form at the time of their enrollment. The Bioethical Committee of the Medical School (Comité de Bioética de la Facultad de Ciencias Médicas de la Universidad Nacional de Cuyo; President: Dr Ramón Piezzi, Vice-President: Dr Edgardo Trimastic) approved our protocol for the collection and manipulation of human sperm samples. All laboratory procedures followed the safety regulations of the Medical School.

### Reagents

Recombinant streptolysin O (SLO) was obtained from Dr Bhakdi (University of Mainz, Mainz, Germany). Spermatozoa were cultured in Human Tubal Fluid media (as formulated by Irvine Scientific, Santa Ana, CA) supplemented with 0.5% bovine serum albumin (HTF media). The rabbit polyclonal anti-NSF (whole serum) and anti-syntaxin1A (whole serum) antibodies as well as the mouse monoclonals anti-α/β-SNAP (clone 77.2, purified IgG) [23] and anti-synaptobrevin2 (clone 69.1, purified IgG) antibodies were from Synaptic Systems (Göttingen, Germany). A mouse monoclonal anti-syntaxin antibody (clone HPC-1, IgG1 isotype, ascites fluid) was from Sigma (St Louis, MO). Horseradish peroxidase-conjugated goat anti-mouse IgG (H+L) was from Kierkegaard & Perry Laboratories, Inc. (Gaithersburg, MD). Horseradish peroxidase-, Cy™3-conjugated goat anti-rabbit and Cy™3-conjugated goat anti-mouse IgGs (H+L) were from Jackson ImmunoResearch (West Grove, PA). *O*-nitrophenyl EGTA-acetoxymethyl ester (NP-EGTA-AM), N,N,N′,N′-tetrakis (2-pyridymethyl) ethylenediamine (TPEN), and BAPTA acetoxymethyl ester (BAPTA-AM) from Molecular Probes were purchased from Invitrogen (Buenos Aires, Argentina). Prestained molecular weight markers were from Boston BioProducts Inc (Worcester, MA). Protein G-sepharose and Ni-NTA-agarose were from GE Healthcare (Buenos Aires, Argentina). All electron microscopy supplies were from Pelco (Ted Pella, Inc., Redding, CA). All other chemicals were purchased from Sigma-Aldrich™ Argentina S.A., Genbiotech, or Tecnolab (Buenos Aires, Argentina).

### Recombinant proteins

A pQE9 (Qiagen GmbH, Hilden, Germany) construct encoding full length, wild type α-SNAP was a kind gift from Dr. S. Whiteheart (University of Kentucky, Lexington, KY). The N-terminal truncated mutant α-SNAP-(160–295) in pQE30 (Qiagen) was generously provided by Dr. A. Morgan and the full length protein bearing the point mutation L294A and cloned in pQE30 (Qiagen) was a kind gift from Dr. R. Burgoyne (both from the University of Liverpool, Liverpool, UK). Plasmid pET28 encoding α-SNAP-M105I was a generous gift from Dr. P. Hanson (Washington University, St. Louis, MO). Plasmids encoding NSF and syntaxin1 (1–262) in pET28a (Stratagene, La Jolla, CA) were generously provided by Dr. D. Fasshauer (Max-Planck Institute for Biophysical Chemistry, Göttingen, Germany). Expression plasmids encoding the light chains of wild type tetanus toxin (TeTx), botulinum toxin C (BoNT/C) and of catalytically dead BoNT/C (BoNT/C-E230A) fused to His_6_ (pQE3, Qiagen) were generously provided by Dr. T. Binz (Medizinische Hochschule Hannover, Hannover, Germany). The expression plasmid encoding amino acids 1–321 of wild type PTP1B fused to His_6_ in pET21b (Stratagene) was kindly provided by Dr. N. Tonks (Cold Spring Harbor Laboratory, Cold Spring Harbor, NY). An expression plasmid pQE80L containing the cDNA-encoding human Rab3A fused to His_6_ was generously provided by Dr. C. López (Cuyo University, Mendoza, Argentina). Purified, recombinant human complexin II fused to glutathione S-transferase (GST) was a kind gift from Dr. C.M. Roggero (Cuyo University, Mendoza, Argentina).

DNAs encoding His_6_-TeTx, wild type α-SNAP and BoNT/C, and BoNT/C-E230A were transformed into *E.coli* XL-1Blue (Stratagene) and protein expression was induced for 3 h at 37°C with 0.6 mM isopropyl-β-D-thio-galactoside (IPTG) (α-SNAP) or overnight at 22°C with 0.1 (BoNT/C and BoNT/C-E230A) or 0.2 (TeTx) mM IPTG. All proteins encoded by cDNAs contained in pET vectors were expressed in *E.coli* BLR(DE3) (Stratagene) by inducing with 0.25 mM IPTG (3 h at 37°C), except PTP1B wild type that was induced with 0.1 mM IPTG overnight at 22°C. DNA encoding α-SNAP-L294A was transformed into *E.coli* BL21 (Stratagene) and protein expression was induced with 0.6 mM IPTG 3 h at 37°C. The cDNA encoding His_6_-Rab3A was transformed into *E.coli* BL21(DE3)pLysS (Stratagene) and expression of recombinant protein was accomplished by incubation with 0.25 mM IPTG overnight at 22°C.

Purification of His_6_-tagged recombinant proteins was carried out under native conditions according to Qiagen's instructions except that the purification buffers contained 20 mM TrisHCl, pH 7.4 instead of 50 mM phosphate, pH 8; NaCl was 200 mM for NSF and 500 mM for the rest; lysis buffer contained 2 mM imidazole, washing buffer contained 8 mM imidazole; and elution buffer contained 400 mM imidazole. 0.5 mM ATP, 5 mM MgCl_2_, 5% glycerol, and 2 mM β-mercaptoethanol were added to all buffers involved in the purification of His_6_-NSF. Syntaxin was extracted from bacterial pellets under denaturing conditions (6 M urea) because most of the protein accumulated in inclusion bodies; afterward, purification was carried out as usual, with syntaxin renaturing during the washing step. Rab3A was prenylated and loaded with GTP-γ-S as previously described [41]. Recombinant protein concentrations were determined by the BioRad Protein assay in 96-well microplates. Bovine serum albumin was used as a standard and the results were quantified on a BioRad 3550 Microplate Reader.

### Human sperm sample preparation procedure. AR assay

Human semen samples were obtained from normal healthy donors. Semen was allowed to liquefy for 30–60 min at 37°C. Following a swim-up protocol to isolate highly motile cells, sperm concentrations were adjusted to 7×10^6^/ml before incubating for at least 2 h under capacitating conditions (HTF, 37°C, 5% CO_2_/95% air). Sperm were washed twice with PBS and resuspended in cold PBS containing 2.1 U/ml SLO for 15 min at 4°C. Cells were washed once with PBS resuspended in ice-cold sucrose buffer (250 mM sucrose, 0.5 mM EGTA, 20 mM Hepes-K, pH 7) containing 2 mM DTT. For AR assays we added inhibitors and stimulants sequentially as indicated in the figure legends, and incubated for 8–10 min at 37°C after each addition. When indicated, we preloaded SLO-permeabilized sperm with photosensitive NP-EGTA-AM before incubating in the presence of inhibitors and/or calcium, carrying out all procedures in the dark. Photolysis was induced after the last incubation by exposing twice (1 min each time) to an U.V. transilluminator (FBTIV-614, Fisher Scientific, Pittsburgh, PA), mixing, and incubating for 5 min at 37°C. Sperm were spotted on teflon-printed slides, air dried, and fixed/permeabilized in ice-cold methanol for 20 sec. Acrosomal status was evaluated by staining with FITC-coupled *Pisum sativum* agglutinin (FITC-PSA, 50 µg/ml in PBS) for 40 min at room temperature followed by a 20 min-wash in water. At least 200 cells were scored using an upright Nikon Optiphot II microscope equipped with epifluorescence optics. Basal (“control”, no stimulation) and positive (“calcium”, 0.5 mM CaCl_2_ corresponding to 10 µM free calcium estimated by MAXCHELATOR, a series of program(s) for determining the free metal concentration in the presence of chelators; available on the World Wide Web at http://www.stanford.edu/~cpatton/maxc.html, Chris Patton, Stanford University, Standord, CA) controls were included in all experiments. Acrosomal exocytosis indices were calculated by subtracting the number of spontaneously reacted spermatozoa from all values and expressing the results as a percentage of the AR observed in the positive control. We only included in our analysis results derived from experiments where the difference between basal and calcium-stimulated conditions was of at least eight percentage points and that produced similar responses. Samples with a level of spontaneously reacted sperm higher than 20% were excluded from our analysis. Data were evaluated using one way ANOVA. The Tukey-Kramer post hoc test was used for pairwise comparisons. Differences were considered significant at the P<0.05 level.

### Transmission electron microscopy

We processed permeabilized human sperm as described for AR assays except that after incubation with 0.5 mM CaCl_2_ to initiate exocytosis, we added 1.6 mM tannic acid and incubated for 5 min at 37°C. Afterwards we fixed the sperm suspensions (5×10^6^/tube) overnight at 4°C or 2 h at room temperature in 2.5% glutaraldehyde in sucrose buffer. Fixed sperm samples were washed twice in PBS (20 min at 4°C each) and postfixed in 1% osmium tetroxide-PBS for 2 h at room temperature, washed three times in PBS (20 min at room temperature each), and dehydrated sequentially with increasing concentrations of ice-cold acetone (twice with 50% for 5 min, once with 70%, once with 80%, and once with 95% all for 15 min, and three times with 100% for 15 min at room temperature). Cells were infiltrated in 1∶1 acetone∶Spurr (a low-viscosity epoxy resin embedding medium for electron microscopy) overnight at room temperature and finally embedded in fresh pure resin overnight at room temperature. Samples were cured 24 h at 70°C. Thin sections were cut (60–80 nm) with a diamond knife (Diatome, Washington, DC) on a Leica Ultracut R ultramicrotome, collected on 200-mesh copper grids and stained with saturated uranyl acetate in methanol plus lead citrate. Grids were observed and photographed in a Zeiss 902 electron microscope at 50 kV. We included negative (not stimulated) and positive (stimulated with 0.5 mM CaCl_2_ in the presence of 20 µM BAPTA-AM or 200 nM complexin to inhibit the final stages of the AR without affecting acrosomal swelling or the number of membrane appositions) controls in all experiments.

### Indirect immunofluorescence

We processed permeabilized human sperm as described for AR assays, adding inhibitors and calcium (stimulant) sequentially as indicated in the figures' keys, and incubating for 8–10 min at 37°C after each addition. Subsequently, we attached 7–10×10^5^ sperm to polylysine coated, 12 mm round coverslips and fixed in 2% paraformaldehyde in PBS for 15 min at room temperature. The fixative was neutralized overnight at 4°C in PBS containing 100 mM glycine. Next, we permeabilized with 0.1% Triton X-100 in PBS for 10 min at room temperature and washed three times with PBS containing 0.1% polyvinylpyrrolidone (PVP, average M.W. = 40,000; PBS/PVP). Nonspecific staining was blocked by incubation in 5% bovine serum albumin in PBS/PVP either with (anti-syntaxin1A immunostaining) or without (anti-synaptobrevin2 immunostaining) 0.2% SDS for 1 h at 37°C. Anti-syntaxin1A (rabbit polyclonal, 1∶50) or anti-synaptobrevin2 (20 µg/ml) antibodies were diluted in 3% bovine serum albumin in PBS/PVP, added to the coverslips, and incubated for 1 h at 37°C in a moisturized chamber. After washing twice for 10 min with 2% saponin in PBS, Cy™3-conjugated goat anti-rabbit or anti-mouse IgGs (5 µg/ml in 1% bovine serum albumin in PBS/PVP) were added and incubated for 1 h at room temperature protected from light. Coverslips were washed six times for 6 min with PBS/PVP. Cells were subsequently stained for acrosomal contents as described before, mounted with 1% propyl-gallate/50% glycerol in PBS containing 2 µM Hoechst 33342 and stored at 4°C in the dark until examination with an Eclipse TE2000 Nikon microscope equipped with a Plan Apo 60×/1.40 oil objective and a Hamamatsu digital C4742-95 camera operated with MetaMorph 6.1 software (Universal Imaging Corp., USA). Background was subtracted and brightness/contrast were adjusted to render an all-or nothing labeling pattern using Image J (freeware from N.I.H.). The presence of immunostaining in the acrosomal region was scored in digital images from at least 10 fields containing ≥200 cells in total. Data were normalized with respect to the percentage of positive cells observed in samples not exposed to neurotoxins (range 38%–47% for syntaxin1 and 34%–40% for synaptobrevin2).

### NSF binding assay

We followed the protocol described by Barnard *et al* [42]. Briefly, 4 µg of recombinant α-SNAPs (wild type and mutants) were incubated in 0.5 ml polypropylene microcentrifuge tubes for 20 min at 4°C. Excess protein was removed and non-specific binding sites blocked by incubating for 2 min at 4°C in 100 µl of washing buffer (50 mM KCl, 1 mM DTT, 25 mM Tris-HCl, pH 7.4) containing 10 mg/ml bovine serum albumin, followed by a similar incubation in washing buffer-1 mg/ml bovine serum albumin. Next, we added 2 µg of recombinant NSF in 20 µl of binding buffer (100 mM KCl, 2 mM EDTA, 0.5 mM ATP, 1 mM DTT, 250 µg/ml soybean trypsin inhibitor, 20 mM Hepes, pH 7.4) and incubated at 4°C for 10 min. After removing the liquid, we washed all tubes with 100 µl of binding buffer, and recovered immobilized proteins by boiling for 5 min in 50 µl of sample buffer. Proteins were resolved on 10% polyacrylamide gels and analyzed by anti-NSF and anti-α/β-SNAP Western blot.

### α-SNAP binding to syntaxin and dissociation by NSF

We followed the protocol described by Hanson *et al* [23] with slight modifications. Briefly, 0.9 µM syntaxin 1–262 was incubated for 2 h at 4°C with the indicated amounts of α-SNAP (wild type and mutants) in binding buffer containing 20 mM Hepes, pH 7.4, 150 mM NaCl, 1 mM DTT, and 0.5% Triton X-100 (final reaction volume 50 µl). After clarification (12,000× g, 4°C, 5 min) and transfer to a fresh tube, we added 1 µl (3.5 µg) of the anti-syntaxin monoclonal antibody and incubated for 1 h at 4°C with gentle shaking. For *in vitro* disassembly experiments, the last step was preceded by an incubation with 0.3 µM NSF plus either ATP or ATP-γ-S (2 mM) for 1 h at 30°C in a thermomixer (Eppendorf, Lobov Científica, Buenos Aires, Argentina) set at 400 rpm. Antibody-protein complexes were recovered on 10 µl (≈ dry volume) of protein G-Sepharose beads and an additional 1 h incubation as before. The beads were collected by centrifugation at 3,000 rpm for 30 sec, washed three times with 1 ml of binding buffer each and bound proteins were eluted by boiling at 95°C for 5 min in 20 µl sample buffer (twice, pooling eluates) and analyzed by anti-α/β-SNAP and anti-syntaxin (HPC-1 antibody) Western blot.

### SDS-PAGE and Western blots

Proteins were separated on tris-glycine-SDS gels [43] and transferred to 0.22 µm nitrocellulose membranes (Hybond, GE Healthcare). Non-specific reactivity was blocked by incubation for 1 h at room temperature with 5% skim milk dissolved in washing buffer (PBS, pH 7.6, 0.2% Tween 20). Blots were incubated with the anti-α/β-SNAP (0.1 µg/ml), anti-NSF (1∶6,000), or anti-syntaxin (HPC-1, 1∶2000) antibodies in blocking solution for 1 h at room temperature. Horseradish peroxidase-conjugated goat anti-mouse-IgG or goat-anti-rabbit IgG were used as secondary antibodies (0.25 µg/ml in washing buffer) with 1 h incubations. Excess first and second antibodies were removed by washing 5×10 min in washing buffer. Detection was accomplished with chemiluminescence systems from either Perkin Elmer (Western Lightning, Migliore Laclaustra, Buenos Aires, Argentina) or Millipore (WBKLS, Biopore, Buenos Aires, Argentina) on a Luminescent Image Analyzer LAS-4000 (Fujifilm, Tokyo, Japan) or exposure to Pierce CL-XPosure Film (Tecnolab). Quantification of signal intensities was carried out with Image J.

## Results

### Exogenous α-SNAP blocks the AR at a stage prior to SNARE assembly in *trans*

**Figure 1:**
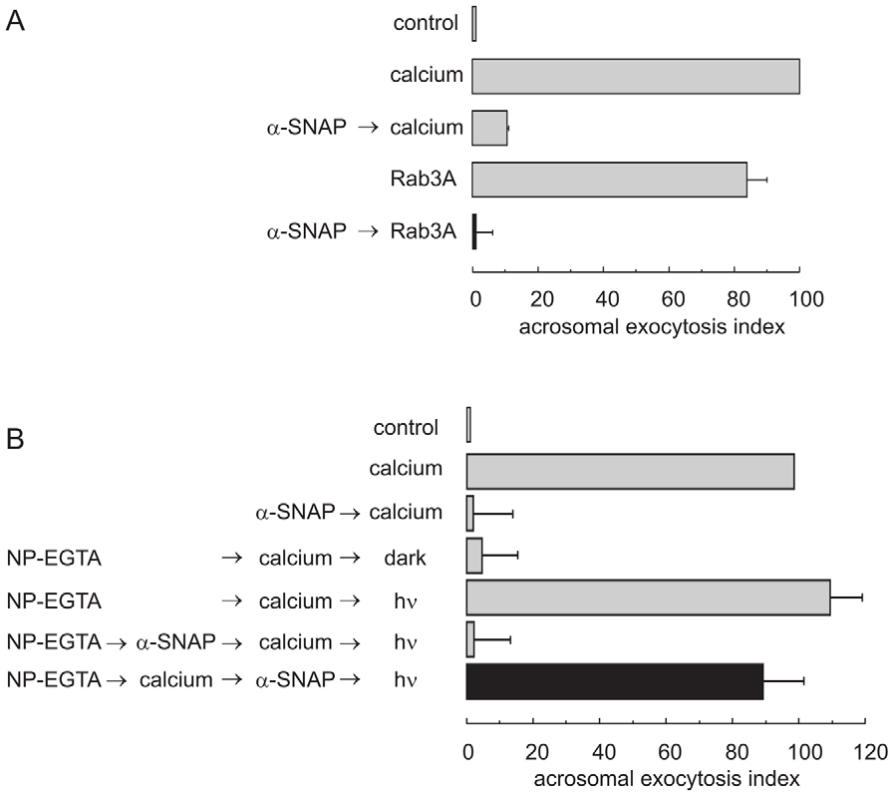
α-SNAP halts human sperm AR before SNAREs assemble in *trans* complexes. *A*, SLO-permeabilized sperm were treated for 15 min at 37°C with 300 nM α-SNAP before initiating acrosomal exocytosis with 300 nM GTP-γ-S-bound Rab3A and incubating at 37°C for 15 min (black bar). Controls (gray bars) included: background AR in the absence of any stimulation (control); AR stimulated by 0.5 mM CaCl_2_ (calcium) or 300 nM GTP-γ-S-bound Rab3A (Rab3A); and AR inhibited by 300 nM α-SNAP (α-SNAP → calcium). Sperm were fixed and stained with FITC-PSA. Exocytosis was evaluated by FITC-PSA binding and data normalized (mean ± S.E.M, three independent experiments) as described under “Materials and Methods.” Actual percentages of reacted sperm for control and calcium ranged between 11–16 and 20–26% respectively. *B*, SLO-permeabilized sperm were loaded with 10 µM NP-EGTA-AM (NP-EGTA) for 10 min at 37°C to chelate intravesicular calcium. Next, the AR was initiated by adding 0.5 mM CaCl_2_ and samples incubated for further 10 min at 37°C. This protocol allows exocytosis to proceed up to the intra-acrosomal calcium-sensitive step, which for SNARE proteins means disassembly of pre-existing *cis* complexes and reassembly of the monomers in loose *trans* arrays. At this point, sperm were treated for 10 min with 300 nM α-SNAP. All these procedures were carried out in the dark. UV photolysis of the chelator was induced at the end of the incubation period (hν) and the samples were incubated for 5 min to promote exocytosis (black bar). Several controls were included (gray bars): background AR in the absence of any stimulation (control); AR stimulated by 0.5 mM CaCl_2_ (calcium); AR inhibited by 300 nM α-SNAP (α-SNAP → calcium); inhibitory effect of NP-EGTA-AM in the dark (NP-EGTA → calcium → dark) and recovery upon illumination (NP-EGTA → calcium → hν); and the inhibitory effect of α-SNAP when present throughout the experiment (NP-EGTA → α-SNAP → calcium → hν). Sperm were stained and the AR scored as described in *A*. Actual percentages of reacted sperm for control and calcium ranged between 13–17 and 25–26% respectively. Shown is the mean ± S.E.M. of at least three independent experiments.

Excess recombinant α-SNAP inhibits the onset of the AR elicited by calcium [29]. To begin to unveil the molecular mechanisms that drive this phenomenon, we asked where in the signaling cascade does this inhibition take place. To this end, we made use of the fact that in human sperm prenylated, GTP-γ-S-loaded, recombinant Rab3A induces a strong exocytotic response that relies on the same fusion machinery characterized for calcium-triggered exocytosis [22], [41]. Specifically, recombinant Rab3A relies on the assembly of SNARE proteins in *trans* complexes to achieve exocytosis [11]. The results depicted in Figure 1A show that pretreatment with α-SNAP inhibited the AR triggered by Rab3A, extending the prior observations made with calcium to a second AR inducer.

To assess when recombinant α-SNAP blocks exocytosis with respect to intracellular calcium release, we applied this protein in combination with the photolabile calcium chelator NP-EGTA-AM. In our SLO-permeabilized human sperm model NP-EGTA-AM crosses the plasma and outer acrosomal membranes, accumulates inside the acrosome, and prevents the AR triggered by all inducers by sequestering intra-acrosomal calcium for as long as the system is kept in the dark. At this point (after loading with NP-EGTA-AM and adding an AR inducer while protecting the tubes from the light), sperm SNAREs are engaged in partially assembled (loose) *trans* complexes that are sensitive to BoNT/s but resistant to TeTx [11]. UV photolysis of NP-EGTA-AM rapidly replenishes the acrosomal calcium pool, resuming exocytosis [17]. In NP-EGTA-AM-loaded permeabilized sperm, recombinant α-SNAP blocked exocytosis when added before — but not after (Fig. 1B, black bar) — the initiation of the AR by calcium. These data indicate that α-SNAP inhibits exocytosis prior to intra-acrosomal calcium efflux. More importantly, they show that recombinant α-SNAP is unable to prevent the AR once SNARE proteins have entered *trans* complexes.

### Binding of α-SNAPs to syntaxin parallels their ability to inhibit the AR

To gain further insights into the mechanisms by which α-SNAP inhibits secretion, we characterized the behaviour of three functionally distinct mutants. The α-SNAP-M105I mutant binds the SNARE complex and allows its disassembly by NSF [32]. α-SNAP-L294A binds syntaxin and NSF but exhibits decreased ability to stimulate NSF's ATPase activity [44]. N-terminal truncated forms of α-SNAP bind NSF *in vitro* when immobilized on plastic surfaces and stimulate its ATPase activity [42], [44], but fail to bind syntaxin when monomeric or engaged in ternary SNARE complexes [23], [26].

**Figure 2:**
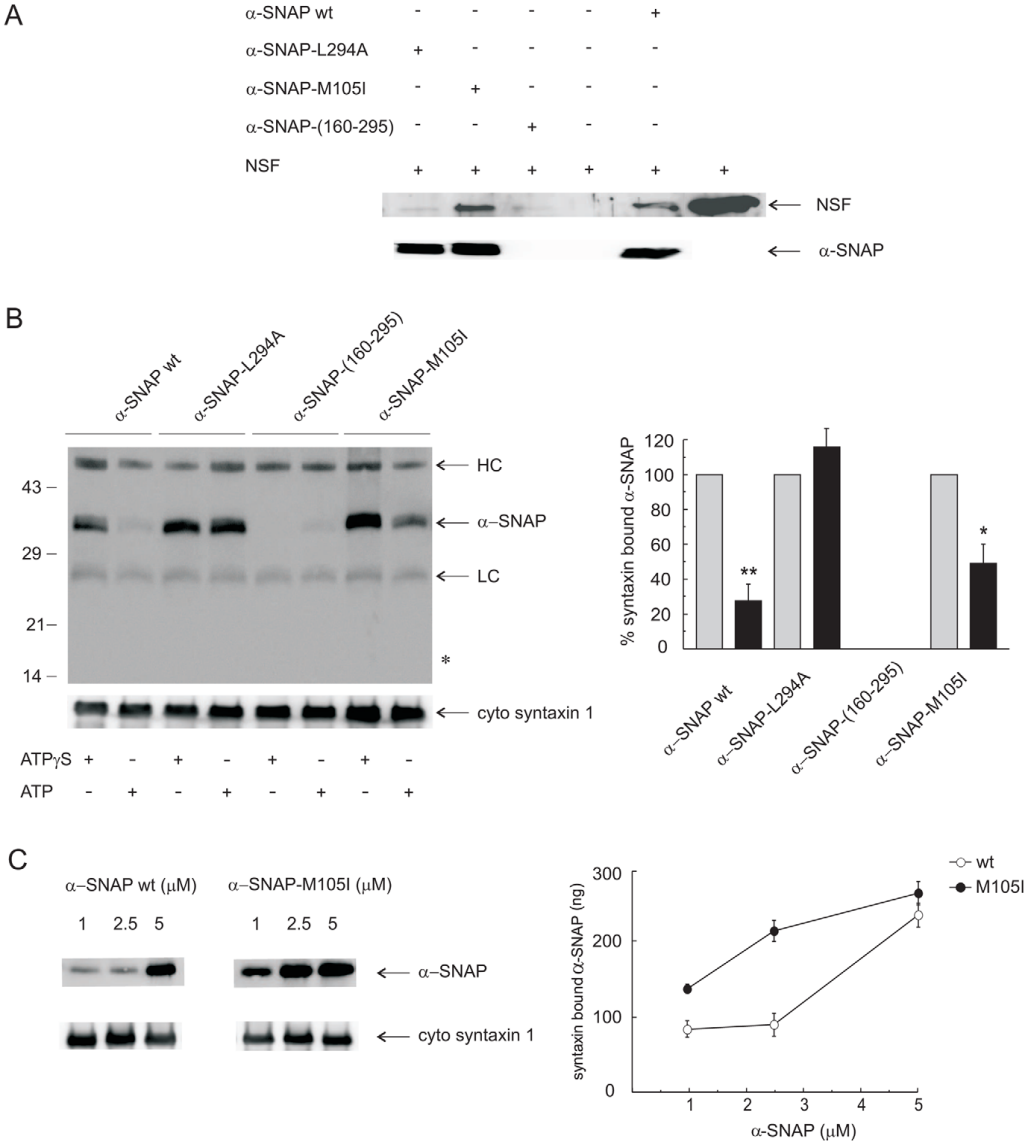
α-SNAP-M105I binds syntaxin with higher affinity than wild type α-SNAP. *A*, 4 µg recombinant α-SNAP (wild type and mutants) were immobilized by binding to the surface of polypropylene microcentrifuge tubes for 20 min at 20°C. Excess protein was removed and non-specific binding sites were blocked as described under “Materials and Methods” before incubating with 2 µg recombinant NSF for 10 min at 4°C. All tubes were then washed and the immobilized proteins were recovered by boiling for 5 min in Laemmli sample buffer. Proteins were resolved on 10% SDS-polyacrylamide gels and analyzed by anti-NSF (top) and anti-α/β-SNAP (loading control, bottom) Western blot. On the far right lane we ran recombinant NSF as a control. Shown is an experiment representative of three repetitions. *B*, *left*, syntaxin1 (0.9 µM) was incubated with 5 µM wild type α-SNAP, 5 µM α-SNAP-(160–295), 5 µM α-SNAP-L294A, or 2.5 µM α-SNAP-M105I in a buffer containing 5 mM MgCl_2_ for 2 h prior to addition of 0.3 µM NSF together with 2 mM ATP or ATP-γ-S. After an additional 1 h at 30°C, syntaxin was collected by inmunoprecipitation as described under “Materials and Methods.” Precipitated protein complexes were separated on 10% SDS-polyacrylamide gels and immunoblotted with the anti-α/β-SNAP (top) or the monoclonal anti-syntaxin1 (bottom) antibodies. LC, immunoglobulin light chain, HC, immunoglobulin heavy chain,* indicates the electrophoretic mobility of α-SNAP-(160–295). *Mr* standards (×10^3^) are indicated on the left. Shown is an experiment representative of three repetitions. *Right*, densitometric analysis of Western blots for α-SNAP (mean ± SEM, n = 3) showing the fraction of α-SNAP coimmunoprecipitated with syntaxin normalized to the amount of syntaxin in each sample. Gray bars, control amount of syntaxin-bound α-SNAP when ATP hydrolysis was prevented (ATP-γ-S lanes, set to 100% for each protein version); black bars, syntaxin-bound α-SNAP after NSF/ATP-driven disassembly expressed as a percentage of the amount precipitated when ATP hydrolysis was prevented. ** p<0.01 for α-SNAP wt ATP vs ATP-γ-S and * p<0.05 for α-SNAP-M105I ATP vs ATP-γ-S (Student's t-test for single group mean); p<0.01 for α-SNAP-M105I ATP vs α-SNAP wt ATP (Student's t-test for unpaired comparison). *C*, Syntaxin1 was incubated with the indicated concentrations of wild type α-SNAP or M105I as in *B*, except that NSF and ATP were omitted, and samples were processed for syntaxin immunoprecipitation and Western blot. Shown is a blot (out of four repetitions) probed for α-SNAP (top) and syntaxin (bottom). *Right*, densitometric analysis of Western blots including that depicted in *C* (mean ±S.E.M., n = 4) showing the maximal amount of α-SNAP coimmunoprecipitated with syntaxin as a function of the initial concentration added to each reaction mixture. Each value was normalized taking into account syntaxin's densitometric signal in the corresponding lane.

First, we investigated the interaction of α-SNAPs — wild type and mutants — with NSF. α-SNAP does not bind NSF in solution but interacts with it when bound to plastic surfaces [42]. Therefore, we immobilized the different α-SNAP proteins on polypropylene tubes, incubated with recombinant NSF, and recovered the proteins by boiling in SDS-sample buffer. Anti-NSF Western blots detected NSF bound to wild type α-SNAP but not to tubes incubated with buffer alone, validating the specificity of the assay. Both wild type α-SNAP and the M105I mutant exhibited high binding to NSF (Fig. 2A). α-SNAP-L294A and α-SNAP-(160–295), on the contrary, bound less NSF than did their wild type counterpart (Fig. 2A), in agreement with published observations [44].

Second, we examined the interaction of the α-SNAP mutants with syntaxin and the lability of the syntaxin-α-SNAP dimers on the presence of activated NSF. To this end, we incubated α-SNAP (wild type and mutants) with the cytosolic domain of syntaxin1; subsequently, we added NSF/ATP/Mg^2+^, and last, we recovered the protein complexes by immunoprecipitation using the anti-syntaxin1 monoclonal antibody. In the control condition we replaced ATP by a non-hydrolyzable analogue. In the presence of ATP-γ-S, wild type α-SNAP and the point mutants were recovered on the protein G-Sepharose beads, indicating that they bound syntaxin (Fig. 2B). The truncated mutant, however, was not found on the beads but on the supernatants of the incubation mixtures (data not shown), consistent with its inability to bind syntaxin. The amount of α-SNAP-L294A that remained bound to the beads upon ATP treatment was similar to that in the ATP-γ-S-treated samples, indicating that NSF was unable to disengage this mutant from syntaxin even under conditions that allowed ATP hydrolysis. When incubated with ATP, NSF was able to release 70% of the wild type but only 40% of the M105I mutant from the beads, even though the wild type was used twice as concentrated (Fig. 2B).When we added α-SNAP-M105I at 5 µM (the same concentration used for the wild type), NSF/ATP failed to dissociate any of it from syntaxin. Even when we added as little as 1 µM mutant, NSF could not release more than 50% (data not shown).

Why was NSF less efficient in dissociating α-SNAP-M105I than wild type from syntaxin? One possibility is that α-SNAP-M105I binds syntaxin with higher affinity. To test this hypothesis we offered 1, 2.5, and 5 µM α-SNAP (wild type and M105I) to syntaxin and immunoprecipitated the protein complexes with the anti-syntaxin antibody. At the lower concentrations tested, the amount of α-SNAP-M105I recovered on the beads was two fold higher than that of the wild type (Fig. 2C). In summary, all the α-SNAP versions tested in this study bound NSF (Fig. 2A and [44]). The three full length proteins also interacted with the cytosolic domain of syntaxin1, with α-SNAP-M105I exhibiting higher affinity than the other two. Recombinant NSF failed to remove α-SNAP-L294A from syntaxin but dissociated both wild type α-SNAP and α-SNAP-M105I, the latter with lower efficiency, possibly due to its higher affinity for syntaxin.

**Figure 3:**
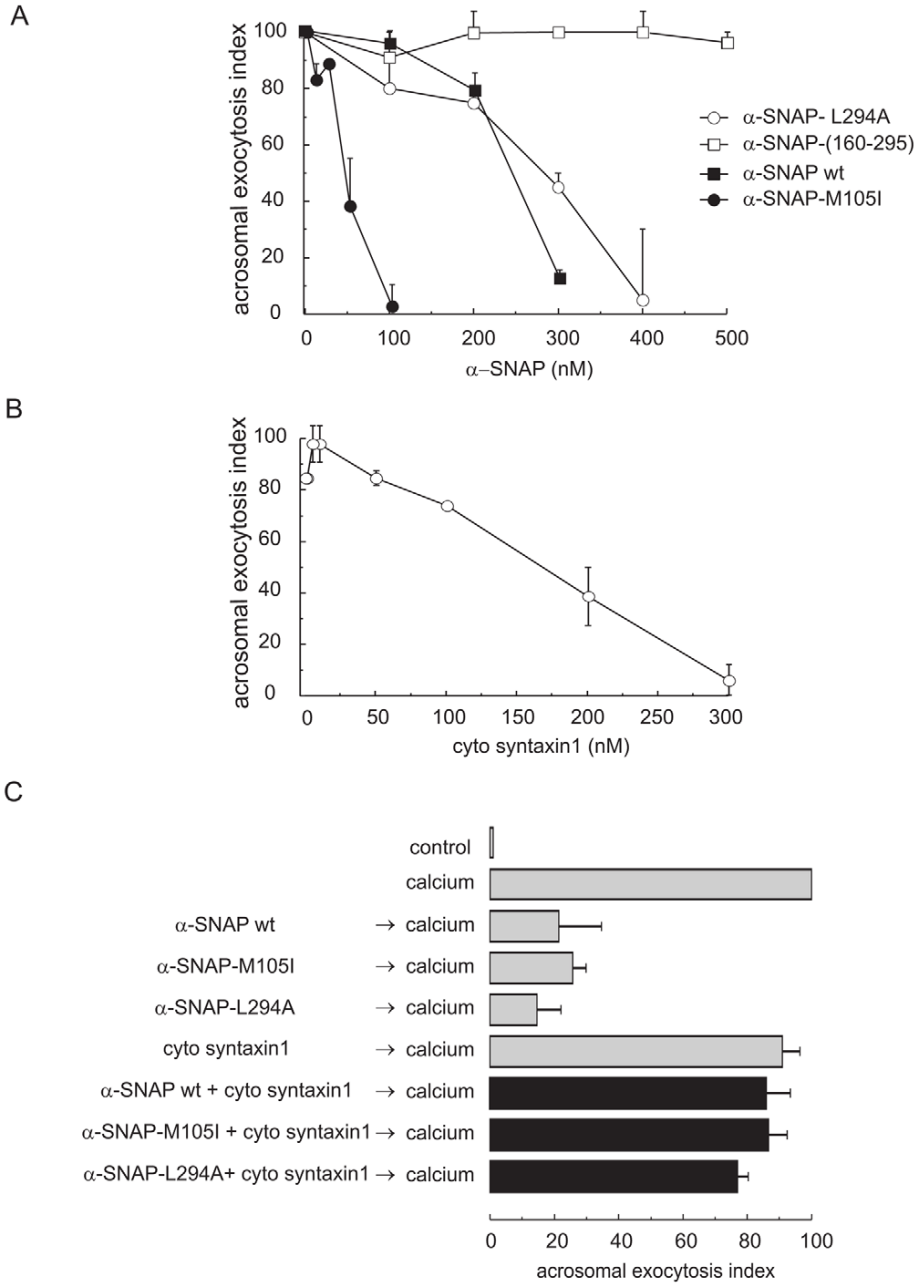
Preincubation with the cytosolic domain of syntaxin1 abolishes the inhibitory effect of α-SNAP on the AR. *A*, SLO-permeabilized human sperm were treated for 15 min at 37°C with increasing concentrations of α-SNAP wild type (closed squares), α-SNAP-(160–295) (open squares), α-SNAP-L294A (open circles), or α-SNAP-M105I (closed circles). Acrosomal exocytosis was initiated by adding 0.5 mM CaCl_2_ and incubating for a further 15 min. Exocytosis was evaluated by FITC-PSA binding and data normalized (mean ± S.E.M. of at least three independent experiments) as described under “Materials and Methods.” *B*, SLO-permeabilized sperm were treated for 15 min at 37°C with increasing concentrations of the cytosolic domain of syntaxin1 (residues 1–262). The AR was subsequently initiated by adding 0.5 mM CaCl_2_ and incubating for 15 min at 37°C. Sperm were stained and the AR scored as in *A*. Shown is the mean ± S.E.M. of at least three independent experiments. *C*, SLO-permeabilized sperm were treated for 15 min at 37°C with 300 nM wild type α-SNAP, 50 nM α-SNAP-M105I or 400 nM α-SNAP-L294A pre-mixed with 100 nM syntaxin1's cytosolic domain (black bars). AR was initiated by adding 0.5 mM CaCl_2_ and incubating for 15 min at 37°C. Several controls were included (gray bars): background AR in the absence of any stimulation (control); AR stimulated by 0.5 mM CaCl_2_ (calcium); AR inhibited by α-SNAPs (α-SNAPs → calcium); AR unperturbed by a low concentration (100 nM) of syntaxin1 (cyto syntaxin1 → calcium). Cells were fixed, acrosomal exocytosis was evaluated by FITC-PSA binding and data were normalized (mean ± S.E.M. of at least three independent experiments) as described under “Materials and Methods”. Actual percentages of reacted sperm for control and calcium ranged between 11–13 and 23–26% respectively.

Next, we characterized the funcional behaviour of α-SNAP and its mutants on sperm exocytosis. When added to permeabilized cells, α-SNAP-M105I caused a dose-dependent inhibition of the calcium-triggered AR, in agreement with previous observations made in human and mouse sperm [34]. α-SNAP-L294A also inhibited the AR with a dose-response curve overlapping that of the wild type version of the protein. In contrast, truncated α-SNAP-(160–295) had no effect even at the highest concentrations tested (Fig. 3A). These results show that recombinant α-SNAPs block the AR with a potency that parallels their ability to bind sperm syntaxin.

Based on the results depicted in Figures 2B and 3A, we reasoned that preincubation with recombinant syntaxin would abolish the capacity of α-SNAPs to block exocytosis. To test this prediction we first examined the effect of the cytosolic domain (residues 1–262) of syntaxin1 on the calcium-triggered AR. Addition of this protein inhibited the calcium-triggered AR in a dose-response fashion (Fig. 3B), likely because it displaced endogenous syntaxin from the *trans* syntaxin-SNAP-25-synaptobrevin ternary complexes necessary for exocytosis. Next, we preincubated α-SNAP (wild type and mutants) at concentrations sufficient to give robust AR inhibition with a low concentration (100 nM) of the cytosolic portion of syntaxin1 before adding them to permeabilized human sperm. The preincubation step attenuated or eliminated the inhibition of calcium-triggered AR seen with the α-SNAP forms alone (Fig. 3C). These results are consistent with the notion that excess exogenous α-SNAP blocks the AR through a mechanism related to its binding to sperm syntaxin.

### NSF reverses α-SNAP-M105I exocytotic block at high NSF/α-SNAP ratios

**Figure 4:**
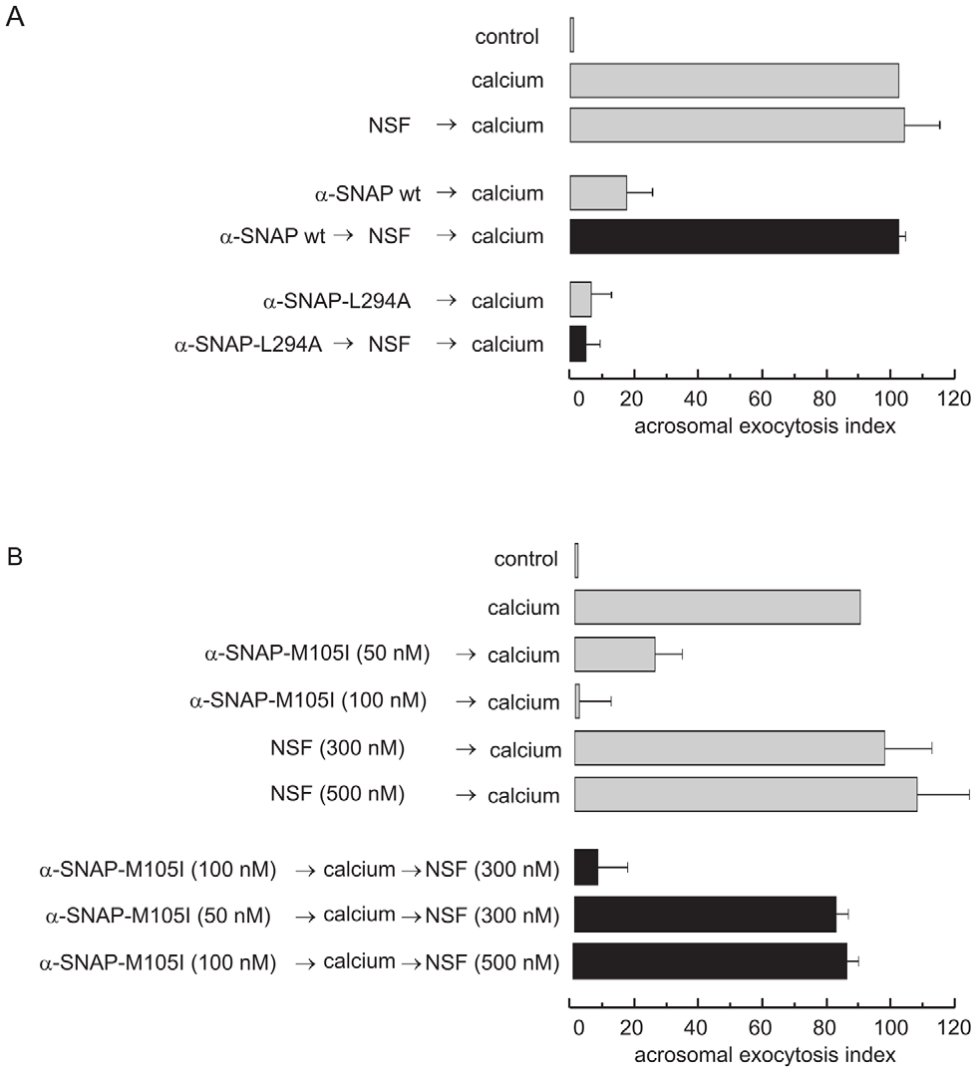
NSF rescues the AR block imposed by the M105I mutant at high NSF/α-SNAP ratios. *A*, SLO-permeabilized sperm were loaded for 15 min at 37°C with 300 nM wild type α-SNAP or 400 nM α-SNAP-L294A followed by 300 nM NSF and the reaction mixtures were incubated at 37°C for 15 min. The AR was initiated by adding 0.5 mM CaCl_2_ and incubating for 15 min at 37°C (black bars). Controls (gray bars) included: background AR in the absence of any stimulation (control); AR stimulated by 0.5 mM CaCl_2_ (calcium); AR inhibited by 300 nM wild type α-SNAP or 400 nM α-SNAP-L294 (α-SNAP wt/L294A → calcium); and AR unperturbed by 300 nM NSF (NSF → calcium). Cells were fixed, acrosomal exocytosis was evaluated by FITC-PSA binding and data were normalized (mean ± S.E.M. of at least three independent experiments) as described under “Materials and Methods.” *B*, SLO-permeabilized spermatozoa were loaded with the indicated concentrations of α-SNAP-M105I for 15 min at 37°C and subsequently challenged with 0.5 mM CaCl_2_ for 10 min at 37°C. NSF was then added as indicated in the labels and incubations continued for an additional 10 min (black bars). Several controls were included (gray bars): background AR in the absence of any stimulation (control); AR stimulated by 0.5 mM CaCl_2_ (calcium); AR inhibited by α-SNAP-M105I (α-SNAP-M105I 50/100 nM → calcium); and AR unperturbed by NSF (NSF 300/500 nM → calcium). Sperm were stained and the AR scored as in *A*. Shown is the mean ± S.E.M. of three independent experiments. Actual percentages of reacted sperm for control and calcium ranged between 12–16 and 26–28% respectively.

The affinity of α-SNAP wild type and mutants for syntaxin (Fig. 2B–C) matched their effectiveness in inhibiting the AR (Fig. 3A). We therefore tested whether the capacity of NSF to release α-SNAPs from syntaxin (Fig. 2B) also paralleled its ability to reverse the exocytotic block. NSF (300 nM) relieved the block imposed by 300 nM wild type α-SNAP on sperm exocytosis (Fig. 4A, middle, black bar and [29]). Note that NSF rescued the inhibitory effect of wild type α-SNAP regardless (α-SNAP wt → NSF → calcium, Fig. 4A). NSF did not, however, rescue the inhibition caused by 400 nM α-SNAP-L294A, which would be expected given that this mutant is unable to stimulate NSF's catalytic activity (Fig. 4A, bottom, black bar). Surprisingly, 300 nM NSF did not reverse the AR blocked by 100 nM α-SNAP-M105I (Fig. 4B). We reasoned that perhaps NSF's activity was overwhelmed by this mutant that exhibited stronger binding to syntaxin1 than the wild type protein (Fig. 2C). To test this possibility we performed similar experiments but either reduced the concentrations of α-SNAP-M105I or increased those of NSF. Three hundred nM NSF reversed the block imposed by submaximal concentrations (50 nM) of α-SNAP-M105I. Likewise, 500 nM NSF achieved exocytosis even in the presence of 100 nM α-SNAP-M105I (Fig. 4B). Taken together, biochemical (Fig. 2B–C) and functional (Figs. 3, 4) data indicate that: i) only recombinant α-SNAP proteins capable of binding syntaxin halted exocytosis; and ii) the ability of NSF to relieve the exocytotic block in permeabilized cells matched its ability to release α-SNAP from syntaxin *in vitro*.

### Neither α-SNAP nor α-SNAP-M105I interfere with sperm SNARE complex disassembly

**Figure 5:**
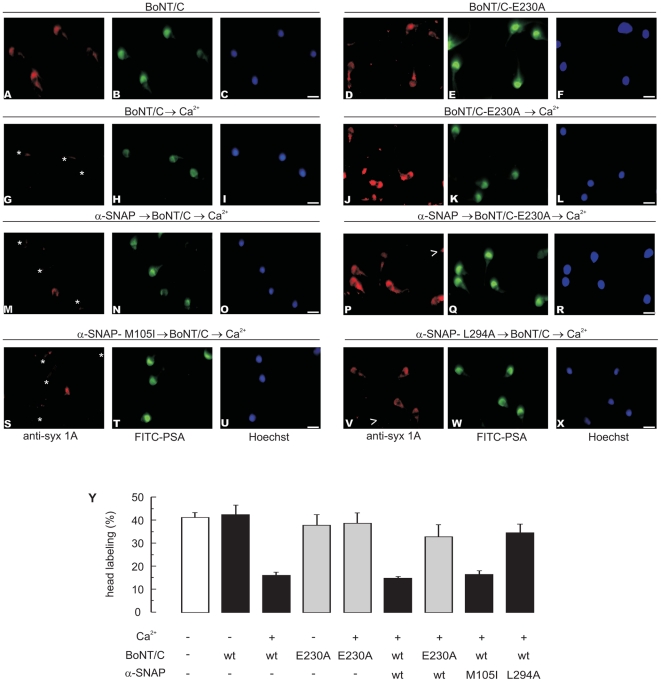
α-SNAP does not interfere with sperm SNARE complex disassembly. Permeabilized spermatozoa were loaded for 15 min at 37°C with 300 nM α-SNAP wild type (*M*, *N*, *O*), 50 nM α-SNAP-M105I (*S*, *T*, *U*), or 400 nM α-SNAP-L294A (*V*, *W*, *X*), subsequently treated with 100 nM BoNT/C and finally activated with 0.5 mM CaCl_2_. The cells were then fixed and triple stained with the rabbit polyclonal anti-syntaxin1A antibody (that recognizes an epitope located in a portion of the molecule released by the toxin; “anti-syx 1A”, red, left panels), FITC-PSA (to differentiate between reacted and intact sperm; green, central panels), and Hoechst 33342 (to visualize all cells in the field; blue, right panels). Notice that reacted sperm were negative for syntaxin1A staining (arrowheads in panels P and V). Asterisks in panels *G*, *M* and *S* indicate cells with intact acrosomes but without syntaxin1 immunostaining due to toxin cleavage. BoNT/C had no effect on syntaxin1A staining in resting sperm (*A*). Labeling in sperm stimulated with calcium was significantly reduced by the wild type (*G*) but not by the catalytically dead (*J*, *P*) BoNT/C. Bars = 5 µm. *Y*, Quantification of the percentage of cells exhibiting syntaxin1 acrosomal staining from three independent experiments (mean ± S.E.M.).

Clostridial toxins impair SNARE assembly into ternary complexes and therefore prevent exocytosis in a variety of cells, including sperm [45], [46]. These neurotoxins exhibit zinc-dependent proteolytic activity that is inactivated by the zinc chelator TPEN. Monomeric SNAREs can be cleaved by botulinum and tetanus toxins, whereas SNAREs engaged in loose (partially assembled at their N-terminal portions) *trans* complexes [47], [48] are only sensitive to BoNTs [11], [49], [50] and those in *cis* complexes are resistant to all [51]. Indirect immunofluorescence as well as exocytosis data indicate that in resting human sperm SNAREs are engaged in toxin-resistant (*cis*) complexes, which are disassembled in response to AR inducers ([11] and this communication). As expected, an anti-syntaxin1 antibody decorated the acrosomal region of permeabilized human sperm cells (quantification shown in Fig. 5Y, open bar); the staining was not affected by pretreatment with the light chain of BoNT/C (Fig. 5A). This neurotoxin is unique because it cleaves two SNAREs, syntaxin and SNAP-25, albeit with different efficiencies [52]. When the toxin was added in combination with calcium, the number of cells exhibiting syntaxin labeling dropped significantly (Fig. 5G), indicating that the initiation of the AR had sensitized this SNARE to BoNT/C. When we substituted wild type BoNT/C for an inactive form of the toxin carrying the point mutation E230A, the percentage of cells exhibiting syntaxin labeling did not decrease (Fig. 5D, J, P), confirming that the drop in the number of stained cells shown on Figure 5G was due to proteolytic cleavage.

Having established that monitoring SNARE proteins integrity by indirect immunofluorescence in the presence of BoNT/C is a suitable assay to determine the evolution of SNAREs configuration (Fig. 5A–L and [11]), we set out to investigate the consequences of adding α-SNAP to the system. We loaded SLO-permeabilized human sperm with wild type recombinant α-SNAP followed by BoNT/C and calcium, and incubated under conditions permissive for toxin cleavage. Fewer cells exhibited syntaxin labeling in the acrosomal region (Fig. 5M), indicating that addition of α-SNAP did not stabilize toxin-resistant *cis* SNARE complexes (as happens in the yeast vacuolar model). The results were similar when we substituted α-SNAP with α-SNAP-M105I (Fig. 5S), which is consistent with the behaviour of this mutant *in vitro*. We tested the validity of these findings with the dominant negative α-SNAP-L294A mutant, which should impair the ability of endogenous NSF to sensitize syntaxin to proteolysis. On running this internal control, we found high acrosomal labeling, indicating that syntaxin was protected from BoNT/C cleavage (Fig. 5V). Furthermore, the results obtained with the L294A mutant reinforce, by means of an independent strategy, the notion that SNARE proteins are engaged in *cis* complexes in resting human sperm and that endogenous α-SNAP is necessary to disassemble them.

**Figure 6:**
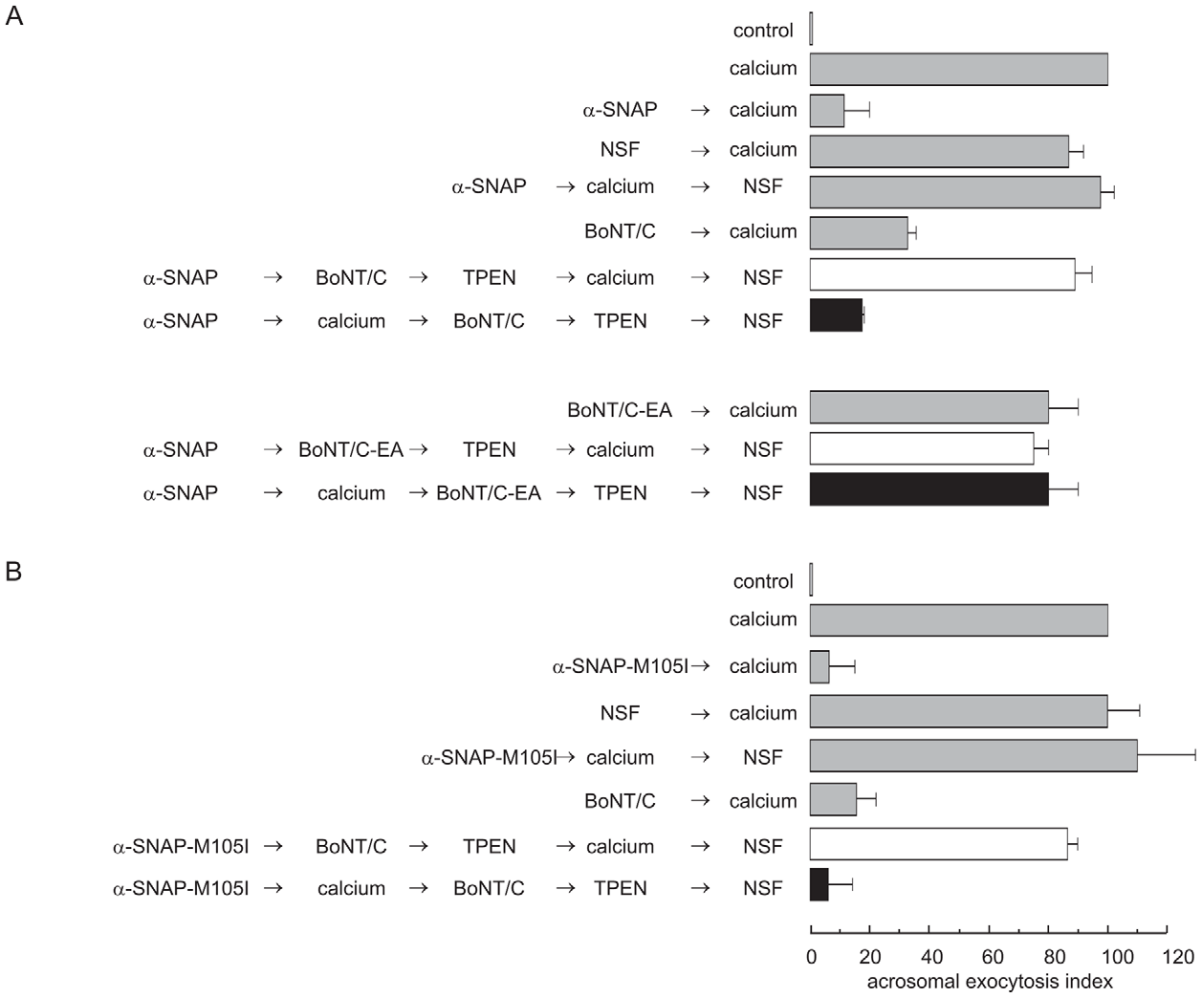
Recombinant α-SNAP halts the AR downstream of SNARE complex disassembly. *A*, to investigate whether α-SNAP impairs *cis* SNARE complex disassembly (which renders syntaxin sensitive to BoNT/C cleavage), we incubated SLO-permeabilized sperm with 300 nM wild type α-SNAP for 10 min at 37°C followed by 0.5 mM CaCl_2_ for an additional 10 min. Subsequently, we added 100 nM light chain of BoNT/C (wild type, *top*, or catalytically dead mutant, *bottom*) and incubated for a further 10 min; we stopped toxin activity with 2.5 µM TPEN for 10 min. At the end of the incubation we released the α-SNAP block with 300 nM NSF for 10 min at 37°C (black bars). To address whether recombinant α-SNAP stimulates endogenous NSF to disassemble sperm *cis* SNARE complexesm even before adding an AR trigger (which leads to NSF's dephosphorylation), we modified the order of addition of reagents as indicated in the figure's labels and incubated as described above (open bars). We included the following controls (gray bars): background AR in the absence of any stimulation (control); AR stimulated by 0.5 mM CaCl_2_ (calcium); AR inhibited by 300 nM α-SNAP (α-SNAP → calcium); AR unperturbed by 300 nM NSF (NSF → calcium); AR inhibited by α-SNAP and rescued by NSF (α-SNAP → calcium → NSF); and AR inhibited by 100 nM wild type (BoNT/C → calcium) but not by the inactive (BoNT/C-E230A → calcium) neurotoxins. Cells were fixed, acrosomal exocytosis was evaluated by FITC-PSA binding and data were normalized (mean ± S.E.M. of at least three independent experiments) as described under “Materials and Methods.” *B*, we conducted experiments identical to those depicted in panel *A*, except that we applied 50 nM α-SNAP-M105I instead of 300 nM wild type α-SNAP. We included the following controls (gray bars): AR inhibited by 50 nM α-SNAP-M105I (α-SNAP-M105I → calcium); AR unperturbed by 300 nM NSF (NSF → calcium); AR inhibited by α-SNAP-M105I and rescued by NSF (α-SNAP-M105I → calcium → NSF); and AR inhibited by 100 nM wild type (BoNT/C → calcium) neurotoxin. Sperm were stained and the AR scored as described in *A*. Shown is the mean ± S.E.M. of at least three independent experiments. Actual percentages of reacted sperm for control and calcium ranged between 7–11 and 19–21% respectively.

We performed the next series of experiments to investigate whether the findings depicted in Figure 5 have a functional correlate in exocytosis. We loaded SLO-permeabilized sperm with wild type recombinant α-SNAP, calcium to initiate the AR, and BoNT/C, and incubated the mixture under conditions in which the toxin could cleave syntaxin; subsequently, we added TPEN to inactivate BoNT/C and NSF to relieve α-SNAP's exocytotic block. Calcium failed to elicit exocytosis under these conditions (Fig. 6A top, black bar). These results indicate once again that the addition of exogenous α-SNAP did not impair the disassembly of *cis* SNARE complexes by sperm NSF because the toxin cut (shown in Fig. 5M), and was subsequently inactivated, prior to the addition of recombinant NSF. When we ran similar experiments while replacing the toxin with the catalytically dead mutant BoNT/C-E230A, we observed neither syntaxin cleavage (Fig. 5P) nor inhibition of the AR (Fig. 6A, bottom, black bar). When we substituted wild type α-SNAP with α-SNAP-M105I, we found that the mutant behaved like the wild type protein and blocked the AR at a stage when syntaxin was susceptible to BoNT/C cleavage (Figs. 5S and 6B, black bar). When we added the same four reagents in the sequence α-SNAPs → toxin → TPEN → calcium → NSF, we observed no inhibition of the AR (Fig. 6, open bars). These results reinforce the notion that BoNT/C could not cleave syntaxin until calcium put the disassembly machinery into motion (Fig. 5A vs G). Our model proposes that before adding an AR trigger, sperm NSF is tyrosine phosphorylated and dormant. We believe this is why recombinant α-SNAPs failed to recruit enough active endogenous NSF to disassemble the native *cis* complexes present in resting cells and sensitize SNAREs to toxins before adding calcium.

**Figure 7:**
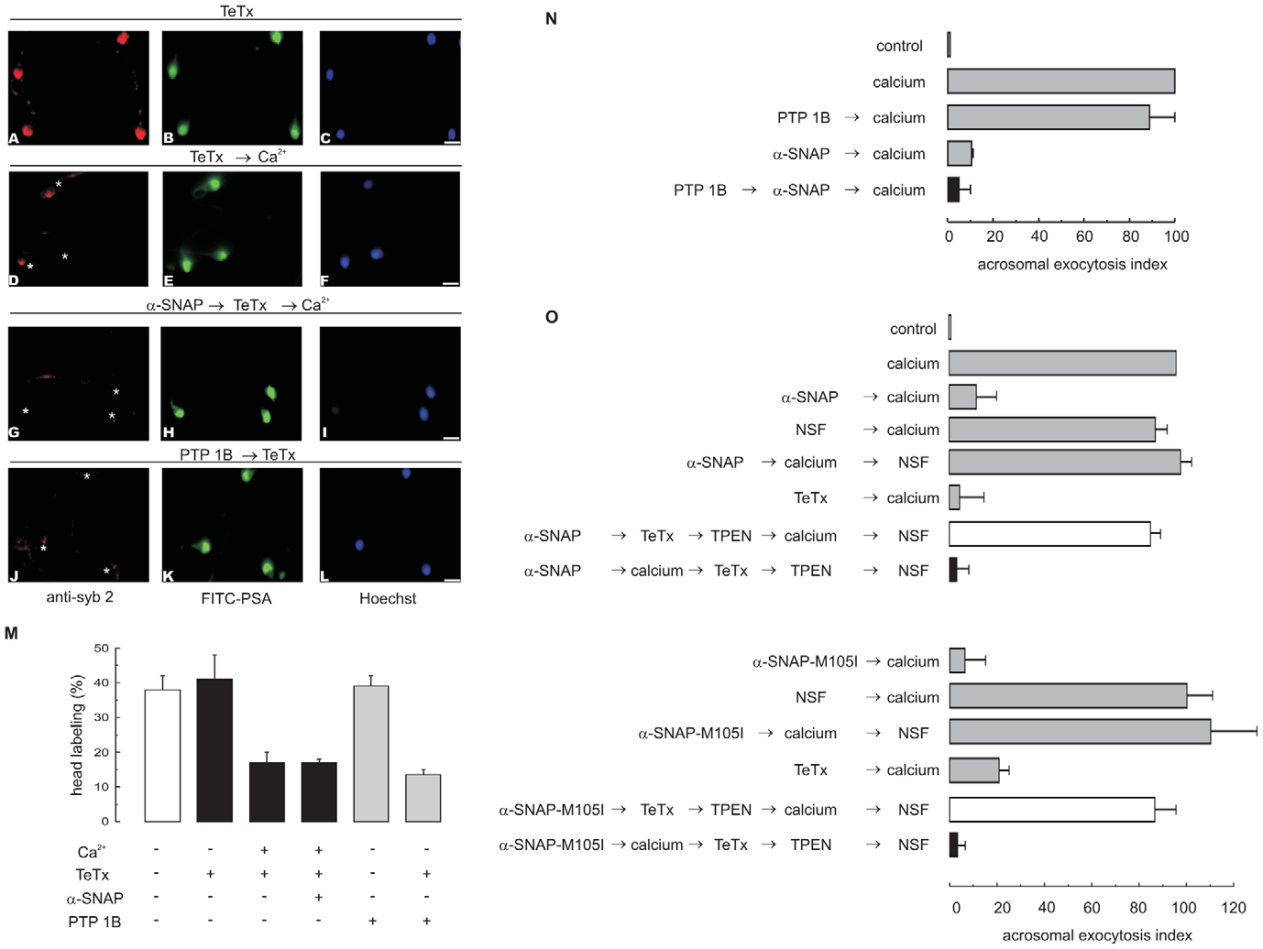
Recombinant α-SNAP halts the AR at a stage when SNAREs are monomeric. *A*, we incubated SLO-permeabilized sperm with 300 nM α-SNAP for 10 min at 37°C followed by 100 nM light chain of TeTx, and 0.5 mM CaCl_2_ for an additional 10 min (*G*, *H*, *I*). To evaluate whether preincubation with PTP1B ultimately leads to *cis* SNARE complex disassembly, we loaded sperm with this phosphatase (27 nM) and treated with TeTx without an exocytotic trigger (*J*, *K*, *L*). We then fixed and triple stained the cells with an anti-synaptobrevin2 antibody (“anti-syb 2”, red, left panels), FITC-PSA (to differentiate between reacted and intact sperm; green, central panels), and Hoechst 33342 (to visualize all cells in the field; blue, right panels). Asterisks in *D*, *G* and *J* indicate cells with intact acrosomes but without synaptobrevin2 immunostaining due to toxin cleavage. TeTx did not cleave synaptobrevin in resting sperm (*A*) but substantially decreased head labeling in sperm stimulated with calcium (*D*). Bars = 5 µm. *M*, percentage of cells showing synaptobrevin2 acrosomal staining from three independent experiments (mean ± S.E.M.). *N*, SLO-permeabilized spermatozoa were loaded with 27 nM PTP1B and incubated for 15 min at 37°C to disassemble *cis* SNARE complexes. Next, we added 300 nM α-SNAP and incubated for a further 15 min at 37°C. Finally, the AR was initiated by adding 0.5 mM CaCl_2_ and incubating for an additional 15 min (black bar). Controls (gray bars) include: background AR in the absence of any stimulation (control); AR stimulated by 0.5 mM CaCl_2_ (calcium); AR inhibited by 300 nM wild type α-SNAP (α-SNAP → calcium); and AR unperturbed by 27 nM PTP1B (PTP1B → calcium). Sperm were fixed and stained with FITC-PSA and the AR scored as described in the legend to Figure 1. Shown is the mean ± range of two independent experiments. *O*, we incubated SLO-permeabilized sperm with 300 nM wild type (top) or 50 nM M105I (bottom) α-SNAP for 10 min at 37°C followed by 0.5 mM CaCl_2_ for an additional 10 min. Subsequently, we added 100 nM light chain of TeTx and incubated for a further 10 min; we stopped toxin activity with 2.5 µM TPEN for 10 min. At the end of the incubation we released the α-SNAPs block with 300 nM NSF for 10 min at 37°C (black bars). To confirm that recombinant α-SNAP does not stimulate endogenous NSF to disassemble sperm *cis* SNARE complexes before adding an AR trigger (to dephosphorylate NSF), we modified the order of addition of reagents as indicated in the figure's labels and incubated as described above (open bars). We included the following controls (gray bars): background AR in the absence of any stimulation (control); AR stimulated by 0.5 mM CaCl_2_ (calcium); AR inhibited by 300 nM α-SNAP (α-SNAP → calcium) and 50 nM α-SNAP-M105I (α-SNAP-M105I → calcium); AR unperturbed by 300 nM NSF (NSF → calcium); AR inhibited by α-SNAPs and rescued by NSF (α-SNAPs → calcium → NSF); and AR inhibited by 100 nM TeTx (TeTx → calcium). Sperm were stained and the AR scored as described in the legend to Figure 1. Shown is the mean ± S.E.M. of at least three independent experiments. Actual percentages of reacted sperm for control and calcium ranged between 10–18 and 21–26% respectively.

We have shown that when added from the beginning of the incubation, recombinant α-SNAP did not interfere with the disassembly of ternary SNARE complexes but halted exocytosis at a stage downstream of this event. We carried out the following experiments in an attempt to confirm these observations by means of a different strategy as well as to take the question of the molecular mechanisms of α-SNAP's inhibition a step further by asking if it is still capable of preventing exocytosis when added after SNARE complex disassembly has taken place. To this end, we made use of recombinant PTP1B. This protein tyrosine phosphatase dephosphorylates sperm NSF, which, together with endogenous α-SNAP, binds and disassembles *cis* SNARE complexes [12], releasing free syntaxin, synaptobrevin and SNAP-25 as reaction products. SNAREs do not spontaneuosly reassemble in *trans*; thus, PTP1B does not trigger exocytosis by itself. The introduction of recombinant PTP1B into permeabilized human sperm sensitized synaptobrevin2 to TeTx cleavage (Fig. 7J, control panels explained below). These results provide direct evidence that PTP1B induces the disassembly of sperm ternary SNARE complexes. Recombinant α-SNAP prevented calcium-triggered exocytosis when added after PTP1B (Fig. 7N). In other words, α-SNAP inhibited the AR when syntaxin was in a monomeric configuration.

### Recombinant α-SNAP inhibits the AR because it sequesters free syntaxin

We introduced TeTx into permeabilized human sperm and performed experiments similar to those depicted in Figure 5 but using the 69.1 monoclonal antibody to assess the integrity of synaptobrevin2. Acrosomal labeling for this SNARE was not affected by incubating unstimulated sperm with TeTx (38% staining in the untreated control vs 40% in TeTx-treated cells; quantification shown in Fig. 7M), consistent with the notion that SNAREs are protected in *cis* complexes in resting sperm. However, a significant decrease in the percentage of cells exhibiting acrosomal staining was observed when toxin loaded sperm were challenged with calcium (Fig. 7D). When we added recombinant α-SNAP to the system, few cells were immunodecorated with the antibody because TeTx had cleaved synaptobrevin (Fig. 7G). These findings confirm those obtained with BoNT/C regarding α-SNAP not interfering with *cis* SNARE complex disassembly.

Data depicted in Figure 1B indicate that α-SNAP blocked the AR at a step prior to assembly of SNARE proteins in *trans*. To gather direct evidence of this mechanism, we resorted to TeTx because it distinguishes between monomeric and complexed synaptobrevin. We applied the same strategy as in the experiments described in Figure 6, but applying TeTx instead of BoNT/C. Briefly, when we loaded SLO-permeabilized sperm with wild type (Fig. 7O top, black bar) or M105I (Fig. 7O bottom, black bar) recombinant α-SNAP, calcium, and TeTx followed by TPEN and NSF, calcium failed to elicit exocytosis. These findings indicate that synaptobrevin was in a monomeric configuration. In other words, α-SNAP did not interfere with *cis* complex disassembly, but it prevented synaptobrevin from entering loose *trans* SNARE complexes, likely because it sequestered syntaxin released from the *cis* complex by NSF and calcium.

To gain further insights into the inhibition of the AR by α-SNAP and rescue by NSF, we created a mathematical model of the biochemical network of reactions driven by these proteins. We used COmplex PAthway SImulator (COPASI) [53] to run simulations that take into account the changes in concentration of all metabolites (Appendix S1). The simulations accurately fit our experimental data (see Figure S1 for details).

### Syntaxin is required to dock the acrosome to the plasma membrane

**Figure 8:**
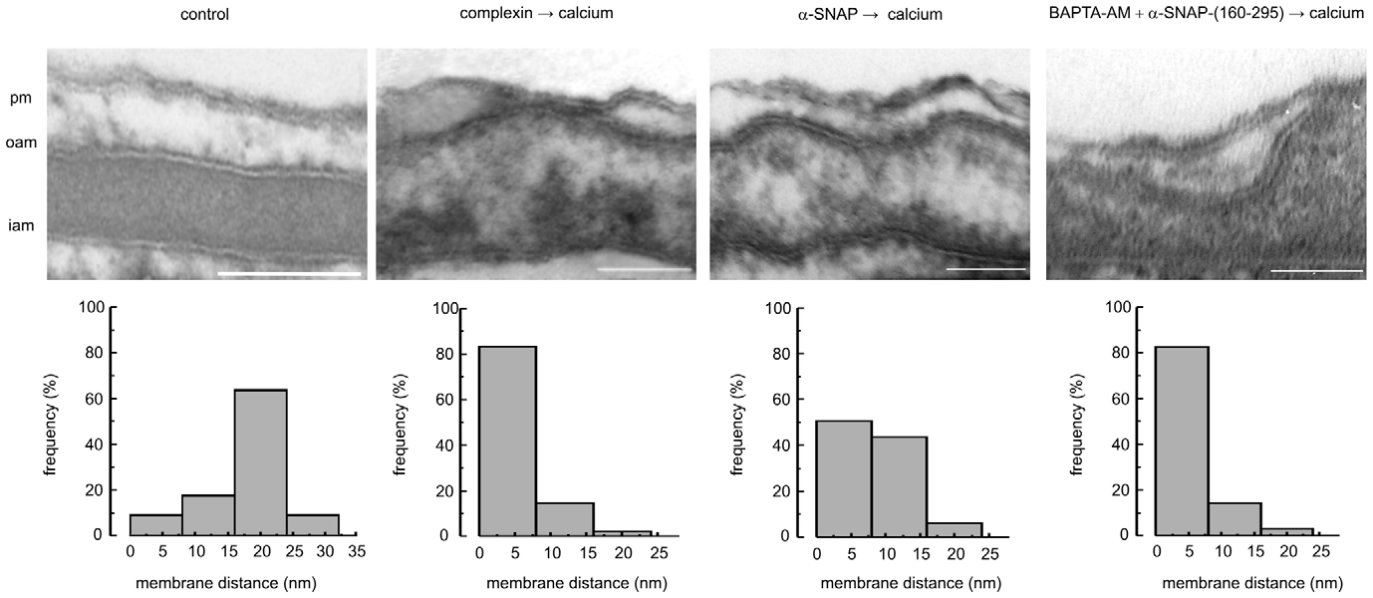
α-SNAP prevents docking of the outer acrosomal to the plasma membrane. To investigate the effect of α-SNAP on the close appositions between the outer acrosomal and plasma membranes required for exocytosis, SLO-permeabilized human sperm were treated for 15 min at 37°C with 500 nM wild type α-SNAP before challenging with 0.5 mM CaCl_2_ (α-SNAP → calcium). We designed the following controls in an effort to validate each aspect of the experiments: i) untreated cells as control for the morphology of unswollen, undocked acrosomes (not shown); ii) 200 nM complexin II for 15 min at 37°C followed by 0.5 mM CaCl_2_ as a control for the morphology of swollen, docked acrosomes (only visible in unreacted cells, hence the choice of late acting AR blockers, “complexin → calcium”); and iii) 500 nM α-SNAP-(160–295) plus 20 µM BAPTA-AM followed by 0.5 mM CaCl_2_ (BAPTA-AM is necessary to prevent acrosomal loss due to exocytosis because the truncated version of α-SNAP is not an AR blocker, “BAPTA-AM → α-SNAP-(160–295) → calcium”). The distance between the outer acrosomal and plasma membranes was measured at the edge of acrosomal invaginations only in images where the membrane bilayers were clearly distinguished. We analyzed 10 cells, 34 contacts, 2 experiments for the untreated control; 16 cells, 48 contacts, 2 experiments, for complexin; 31 cells, 99 contacts, 3 experiments for α-SNAP; and 19 cells, 63 contacts, 2 experiments for α-SNAP-(160–295). Distance distributions are plotted as histograms below each electron micrograph and compared using the Kolmogorov-Smirnov test for two sets of data. Three different distribution patterns were identified: one corresponding to the distance distribution in untreated cells (control); one in cells treated with complexin or BAPTA-AM plus α-SNAP-(160–295); and one in cells treated with wild type α-SNAP (p<0.01 compared with the histogram of cells treated with complexin). pm = plasma membrane; oam = outer acrosomal membrane. Scale bars = 100 nm.

Docking, a strong — and spatially tight — interaction of two membranes engaged in fusion is a mandatory step that precedes bilayer mixing [39]. In the AR model, docked acrosomes approach the plasma membrane with separations less than 10 nm, whereas the undocked acrosomes are evenly separated (18 nm) from the cell membrane [38]. For the next series of experiments, we treated permeabilized human sperm as stated in Figure 8's legend and evaluated the docking status of the acrosomes by transmission electron microscopy, recording distances between the plasma and acrosomal membranes and plotting them as histograms. In cells not challenged to undergo the AR, we found neither acrosomal swelling nor membrane appositions (Fig. 8, control). Instead, there was a high frequency of contacts longer than 16 nm (25 out of 34 contacts, 74%) and a low frequency of shorter contacts (9 out of 34 contacts, 26%). This profile represents the unswollen, undocked state typical of resting cells. In contrast, when we challenged sperm with calcium but prevented acrosomal loss with the late-acting inhibitor complexin II, we found a very high frequency (40 out of 48 contacts, 83%) of very close appositions (between 0 and 8 nm). We encountered fewer longer distance contacts (7 of 48 between 8 and 16 nm, 14%; 1 of 48 between 16 and 20 nm apart, 2%). This profile represents the docked state (Fig. 8, complexin → calcium). When we introduced wild type α-SNAP prior to the calcium challenge, the frequency of very close (0–8 nm) contacts diminished (50 of 99, 51%) whereas that of contacts in the 8–16 nm and over 16 nm ranges augmented (43 of 99, 43% and 6 of 99, 6%) (Fig. 8, α-SNAP → calcium). We analyzed the behavior of the truncated mutant α-SNAP-(160–295) after initiating the AR with calcium but preventing acrosomal loss with the late-acting inhibitor BAPTA-AM, which does not exhibit a docking phenotype *per se* (data not shown). We observed high frequency of short-distance contacts (52 of 63, 83% between 0 and 8 nm) and the histogram was almost identical to that of the complexin control (Fig. 8, BAPTA-AM → α-SNAP-(160–295)→calcium). The percentage of swollen acrosomes was around 25% in all cells treated with calcium, indicating that α-SNAP perturbs an exocytotic stage operating downstream of swelling. These data show that exogenous full length α-SNAP impairs the docking of the acrosome to the plasma membrane elicited by an AR trigger. Because the N-terminal truncated mutant unable to interact with syntaxin did not exhibit an altered docking behaviour, we conclude that α-SNAP needs to bind this Q-SNARE in order to prevent docking. In other words, syntaxin is necessary to accomplish the docking step during sperm exocytosis.

## Discussion

Very similar versions of the proteins required for exocytosis and its regulation play the same roles in virtually all membrane fusion models, including sperm's acrosomal discharge. The AR depends on Rab3A activation, α-SNAP/NSF, synaptotagmin VI, complexin, and SNAREs (toxin-sensitive members of the synaptobrevin, syntaxin, and SNAP-25 families); it also requires cAMP and Epac [6], [7], [16], [54]–[60] (see Fig. S2 for a simplified model). The activity of NSF is repressed by tyrosine phosphorylation in resting sperm. Once exocytosis begins, PTP1B dephosphorylates NSF and derepresses its activity [12]. Aided by α-SNAP, NSF unpairs *cis* SNARE complexes to make monomeric SNAREs available for subsequent *trans* pairing and fusion. The AR is sensitive to dominant negative mutants of NSF that cannot bind or hydrolyze ATP [14], to NSF-sequestering antibodies [21], [29], and to reagents that prevent NSF tyrosine dephosphorylation [12]. Sperm exocytosis is also sensitive to anti α-SNAP antibodies, which demonstrates the essential role of this protein [21], [29]. Conversely, an excess of recombinant α-SNAP prevents the onset of the AR [29]. As is the case with three other fusion scenarios: PC12 cells, reconstituted liposomes [28]g1033 , and yeast vacuoles [30], α-SNAP's inhibitory effect on the AR is reversed by NSF (this report and [29]). It has recently been shown that an excess of recombinant α-SNAP inhibits exocytosis in PC12 cells by binding to syntaxin's SNARE motif and preventing subsequent SNARE pairing [28]. Here we report that recombinant α-SNAP utilizes an identical molecular mechanism to prevent acrosomal release in human sperm. Furthermore, we show that by halting exocytosis at this stage, α-SNAP impaired the docking of the secretory vesicle to the plasma membrane (Fig. 8). Interestingly, if added after syntaxin had entered *trans* SNARE complexes, α-SNAP no longer blocked the AR (Fig. 1B), reinforcing the notion that the inhibition depends on access to monomeric syntaxin. Data depicted on Figures 5M, 6A, 7G and O indicate that addition of α-SNAP to permeabilized cells did not interfere with *cis* SNARE complex dissociation, catalyzed by endogenous NSF in response to AR triggers. This dissociation reaction produces free syntaxin, which also binds α-SNAP. The interaction of recombinant α-SNAP with monomeric syntaxin, rather than that with *cis* SNARE complexes, was responsible for the exocytotic block. When we entered the reactions representing these protein-protein interactions into COPASI, it predicted dose-response curves that fitted accurately the experimental data (Fig. S1 A, C and E).

The notion that vesicles must attach, even transiently, to the compartment they are going to fuse with gave origin to the concept of docking. Because several labs working in different models have developed a variety of assays to assess docking, there is substantial disparity within the topic; this problem is compounded by disagreements in the nomenclature employed to define it (for reviews see [39], [61], [62]). Docking was first described through studies of fixed samples using transmission electron microscopy. In cells specialized for regulated exocytosis there is a pool of docked vesicles (that is, located at distances <25 nm from the plasma membrane) even before triggering, a phenomenon not seen in other fusion processes; it has been proposed that exocytosis of vesicles from this pool accounts for a fast component of secretion, kinetically described as the readily releasable pool. Sperm contain a single, very large dense-core secretory granule. In resting human cells, this granule's membrane is parallel to the plasma membrane, separated ≈18 nm from it. Based on morphological and biochemical evidence, we do not believe there is a pool of cells with readily releasable (pre-docked) acrosomes prior to exposure to an AR inducer, which is consistent with the slow kinetics of acrosomal content discharge. This might be different in boar sperm, where a recent report shows a tight approximation of acrosomal and plasma membranes during capacitation [63]. Once exocytosis begins, the acrosome swells, approaching the plasma membrane until numerous contact zones (distances 0–8 nm) develop; eventually fusion pores open in these zones. We can detect membrane appositions when the experimental conditions are such that syntaxin, synaptobrevin, and SNAP-25 engage in partially (N-terminal) assembled loose *trans* complexes (SNAREs remain in this configuration for as long as the efflux of calcium from the intra-acrosomal store is prevented [11]). An important finding we wish to report here is that addition of recombinant α-SNAP hinders the docking step during the exocytotic cascade (Fig. 8), likely because it prevents monomeric syntaxin from entering *trans* SNARE complexes. In other words, free syntaxin is required for the docking of the acrosome to the plasma membrane.

A 3D model structure of α-SNAP shows residue 105 lying on the back face of the protein, the region responsible for interaction with NSF. Mutations in this region do not alter the ability of α-SNAP to bind SNARE complexes or impair its capacity to stimulate NSF's disassembly activity [64]. Far ultraviolet circular dichroism spectra, synaptic SNARE binding properties, and NSF-catalyzed SNARE complex dissociation kinetics are indistinguishable between the M105I mutant and the wild type protein [32]. Likewise, our results indicate that α-SNAP-M105I does not interfere with the disassembly of ternary SNARE complexes in human sperm (Figs. 5S, 6B and 7O). Yet, *hyh* mice are subfertile due to defective sperm exocytosis caused by the M105I mutation on α-SNAP; treatment with wild type — but not M105I — α-SNAP rescues the AR in sperm from these animals [34]. We have observed that: i) α-SNAP-M105I bound free syntaxin with much higher affinity than did the wild type protein (Fig. 2 B–C) ; ii) recombinant α-SNAP-M105I was a more potent AR inhibitor than the wild type α-SNAP (Fig. 3A); and iii) higher concentrations of NSF were necessary to release mutant than wild type α-SNAP from syntaxin, to allow syntaxin to enter the fusion acceptor complex, and to reverse the exocytotic block (Figs. 4B and S1 B, D and F). Based on all these data, we would like to suggest that the fertility problem in the *hyh* animals might be a consequence of their mutated version of α-SNAP binding monomeric syntaxin with such high affinity that it overwhelms the dissociating activity of sperm's NSF. As a matter of fact, even though the level of α-SNAP protein present in sperm from *hyh* males is identical to that from wild type mice, the amount of NSF is slightly increased [34], as if the cells are trying to compensate for the fertility defect by synthesizing more NSF. We plan to explore this issue in the future by analyzing the exocytotic response of *hyh* sperm supplemented with purified NSF. If our hypothesis is correct, these experimental conditions should be able to rescue the fertility defect.

α-SNAP/NSF disassemble fusion-incompetent *cis* SNARE complexes, yielding monomeric syntaxin, synaptobrevin, and SNAP-25. These proteins exhibit a high propensity to reassemble and it would seem likely that, given their proximity, they would re-engage in *cis* complexes. Because the availability of free SNAREs to form fusion-competent *trans* complexes relies on maintaining low levels of *cis* complexes, it is tempting to speculate about the existence of molecular mechanisms that have evolved to avoid a futile “reassembly-in-*cis*” pathway that interferes with fusion. One such mechanism proposed several years ago is based on the interaction *in vitro* between closed monomeric syntaxin1 (e.g when its N-terminal three helix bundle or Habc domain binds its own SNARE motif) and the synaptic protein Munc18-1 (reviewed in [3], [65]–[67]). It is believed that this interaction interferes with SNARE complex assembly; therefore, if it took place *in vivo*, it would constitute a mechanism to regulate the availability of free syntaxin in cells. It has been shown many years ago that binding of Munc18-1 and α-SNAP to syntaxin are mutually exclusive [26], but this notion has not been tested rigorously ever since. The pathway catalyzed by α-SNAP analyzed in this report would be a likely candidate for maintaining low levels of *cis* complexes if it existed in unperturbed cells containing the endogenous fusion machinery. α-SNAP would assist NSF in disassembling *cis* SNARE complexes and would subsequently bind syntaxin, maintaining it temporarily unavailable. When the conditions were mature, NSF would release α-SNAP and syntaxin would continue the pathway to enter *trans* complexes. Based on results from experiments carried out with sperm from *hyh* animals complemented by those described in this article employing the M105I mutant, we would like to propose that such a mechanism might actually exist.

## Supporting Information

Figure S1 **Modelling of the AR inhibition by α-SNAP and rescue by NSF; comparing the behaviour of wild type α-SNAP and α-SNAP-M105I.**
(DOC)

Figure S2 **Working model for the biochemical cascades driving the late stages of the human sperm AR.**
(DOC)

Appendix S1 **The COPASI model for binding of wild type α-SNAP and α-SNAP-M105I to sperm** ***cis*** **SNARE complexes and free syntaxin; dissociation by NSF.**
(CPS)
