# Supplementary material for: α-SNAP Prevents Docking of the Acrosome during Sperm Exocytosis because It Sequesters Monomeric Syntaxin
Source: PLoS One. 2011 Jul 18;6(7):e21925. doi: 10.1371/journal.pone.0021925 (PMC3138754; doi:10.1371/journal.pone.0021925)
Supplement: Figure S1 — Modelling of the AR inhibition by α-SNAP and rescue by NSF; comparing the behaviour of wild type α-SNAP and α-SNAP-M105I. (DOC) [file pone.0021925.s001.doc]

**SUPPORTING INFORMATION RESULTS**

**Modelling of the AR inhibition by α-SNAP and rescue by NSF**

Complex biochemical processes driven by biological networks can be more easily understood with the help of mathematical tools such as simulation and modelling. Thus, with the purpose of gaining further insights into the mechanisms described so far, we used COmplex PAthway SImulator (COPASI) (1). We fed the program with known rate parameters taken from the literature and specified the initial conditions based on our own experimental data, assuming that the total amount of α-SNAP during the window of time analyzed was conserved (no protein synthesis or degradation; see “Appendix S1” provided as Supporting Information). With this information, COPASI solves the concentration changes for all implicated metabolites, which, in our case, are the moieties represented in the following five reactions:

Reaction 1 (named “SNARE-A” in COPASI) assembly of ternary SNARE complexes

syntaxin + synaptobrevin + SNAP-25 → SNARE complex

Reaction 2 (named “aS-SNARE” in COPASI): binding of α-SNAP to ternary SNARE complexes

α-SNAP + SNARE complex → α-SNAP-SNARE complex

Reaction 3 (named “SNARE-D” in COPASI): disassembly of α-SNAP-SNARE complex by NSF

α-SNAP-SNARE complex + 6NSF → syntaxin + synaptobrevin + SNAP-25 + α-SNAP + 6NSF

Reaction 4 (named “aS-Stx” in COPASI): binding of α-SNAP to syntaxin

α-SNAP + syntaxin → α-SNAP-syntaxin

Reaction 5 (named “aSStx-D” in COPASI): disassembly of α-SNAP-syntaxin complex by NSF

α-SNAP-syntaxin + NSF → syntaxin + α-SNAP + NSF

Data depicted in Figure S1 show the variations predicted by COPASI in the amount of *cis* SNARE complexes, free syntaxin and syntaxin-α-SNAP dimers caused by increasing concentrations of α-SNAP wild type and the M105I mutant in the absence or presence of added NSF. The simulations fit accurately our experimental data and predict that neither of these two α-SNAPs interferes with *cis* SNARE complex disassembly. Syntaxin released at this reaction, however, does not remain free but binds and forms stable dimers with α-SNAPs (this happening at lower α-SNAP-M105I than wild type α-SNAP concentrations). Dimer assembly is responsible for the exocytotic block imposed by these two proteins (Fig. 3). NSF disengages wild type α-SNAP-syntaxin dimers more efficiently than α-SNAP-M105I-syntaxin dimers. These predictions are consistent with experimental data (Figs. 2B and 4B) that show that NSF rescues exocytosis at a lower NSF/α-SNAP ratio with the wild type than with the mutant protein.

**LEGEND TO FIGURE S1**. **Modelling of the AR inhibition by α-SNAP and rescue by NSF; comparing the behaviour of wild type α-SNAP and α-SNAP-M105I**. Five reactions mediating the fraction of syntaxin distributed among three different species (engaged in *cis* SNARE complexes, free, and associated with α-SNAP) were modeled in COPASI (which is free for academic use and available at [www.copasi.org](http://www.copasi.org/); the file named “Appendix S1” is provided as Supporting Information). Kinetic parameters for the interaction of wild type α-SNAP and the M105I mutant with SNARE complexes and monomeric syntaxin were adjusted to the apparent dissociation constants calculated from binding experiments like the one shown in Figure 2 and from references in the literature (2-4). Initial SNARE complex concentration was set to 1 nM and monomeric SNAREs concentrations to zero each. NSF was set to 1 nM (referred to as endogenous NSF, light traces) or to 250 nM (dark traces; larger NSF concentrations could not be assessed because the simulations crashed). We tested different α-SNAP initial concentrations in the 0 to 500 nM range. For each simulation, the system was allowed to progress from the initial conditions to the equilibrium. Final concentrations were plotted for the ternary SNARE complex (synaptobrevin-syntaxin-SNAP-25, 1:1:1; A, B), free syntaxin (C, D), and the α-SNAP-syntaxin dimer (E, F). *Cis* SNARE complex disassembly was achieved by both endogenous and exogenous NSF at low α-SNAP concentrations and was not inhibited by high concentrations of wild type (A) or mutant α-SNAP (B). Wild type α-SNAP bound (E, light green)  and therefore decreased  the amount of free syntaxin (C, light blue). Free syntaxin is necessary for the assembly of the SNAP-25-syntaxin acceptor complex; thus, by predicting its disappearance due to transformation into the α-SNAP-syntaxin dimer at high α-SNAP concentrations, the modelling reflects the inhibition of exocytosis we observed in functional assays (Figs. 3 and 4). Significant amounts of free syntaxin were available in the presence of excess NSF even when large amounts of wild type α-SNAP were included in the simulation (C, dark blue). Thus, the simulation reflects the rescue of the exocytotic block by NSF determined in functional assays (Fig. 4A). The M105I mutant was significantly more potent than the wild type protein in depleting the pool of free syntaxin (D, light blue). The addition of exogenous NSF released enough monomeric syntaxin to overcome the exocytotic block only at low concentrations of the mutant (D, dark blue). These two predictions are in complete agreement with functional data (Figs. 2B and 4B). As the concentrations of wild type or mutant α-SNAP increased, most of syntaxin was sequestered in syntaxin-α-SNAP complexes (E, F). Yet, as long as NSF guaranteed a free syntaxin concentration (e.g. ≥ 0.2 nM in the model), exocytosis could be accomplished.

REFERENCE LIST

1. Hoops, S., Sahle, S., Gauges, R., Lee, C., Pahle, J., Simus, N., Singhal, M., Xu, L., Mendes, P., and Kummer, U. (2006) *Bioinformatics.* **22,** 3067-3074

2. Marz, K. E., Lauer, J. M., and Hanson, P. I. (2003) *J. Biol. Chem.* **278,** 27000-27008

3. McMahon, H. T. and Sudhof, T. C. (1995) *J. Biol. Chem.* **270,** 2213-2217

4. Kee, Y., Lin, R. C., Hsu, S. C., and Scheller, R. H. (1995) *Neuron.* **14,** 991-998

**Figure S1**
