# Supplementary material for: α-SNAP Prevents Docking of the Acrosome during Sperm Exocytosis because It Sequesters Monomeric Syntaxin
Source: PLoS One. 2011 Jul 18;6(7):e21925. doi: 10.1371/journal.pone.0021925 (PMC3138754; doi:10.1371/journal.pone.0021925)
Supplement: Figure S2 — Working model for the biochemical cascades driving the late stages of the human sperm AR. (DOC) [file pone.0021925.s002.doc]

**Figure S2**

**LEGEND TO FIGURE S2**. **Working model for the biochemical cascades driving the late stages of the human sperm AR.** Calcium enters the cell from the extracellular milieu when channels open in the plasma membrane in response to physiological inducers or through the SLO-generated pores. Once into the cytosol, calcium activates sAC, which synthesizes cAMP [1]. These reactions are accompanied by the swelling of the acrosome [2]. Epac-cAMP indirectly activates Rab3A, tethering the acrosome to the plasma membrane [1]. A reaction taking place during or as a consequence of tethering initiates the activation and/or recruitment of PTP1B, which in turn dephosphorylates and activates NSF. Next, NSF/α-SNAP convert pre-existing *cis* SNARE complexes into monomeric syntaxin, synaptobrevin and SNAP-25 [3]. Aided by a number of factors, these proteins assemble in *trans* complexes, docking the plasma and outer acrosomal membranes to each other [2,4,5]. Epac-cAMP drives a parallel signaling pathway, initiated when it directly activates Rap1. In turn, active Rap1 stimulates the synthesis of IP_3_ through the stimulation of a PLC activity [1]. IP_3_ elicits the efflux of calcium from IP_3_-sensitive stores (the acrosome being one of them and likely the most relevant for the AR) [6]. Calcium released from the acrosome through IP_3_-sensitive channels reaches the appropriate local concentrations to bind synaptotagmin [4]. Both assembly of *trans* SNARE complexes and intra-acrosomal calcium efflux are required for the AR. Steps affected by recombinant proteins, toxins, and intra-acrosomal calcium blockers are indicated in blue. Solid arrows mean there is one step between the terms connected, whereas dashed arrows mean that the number of steps is unknown. PM, plasma membrane; OAM, outer acrosomal membrane. This Figure is a modification of the one originally published in [1].

Reference List

1. Branham MT, Bustos MA, De Blas GA, Rehmann H, Zarelli VE et al. (2009) Epac activates the small G proteins Rap1 and Rab3A to achieve exocytosis. J Biol Chem 284: 24825-24839.

2. Zanetti N, Mayorga LS (2009) Acrosomal Swelling and Membrane Docking Are Required for Hybrid Vesicle Formation During the Human Sperm Acrosome Reaction. Biol Reprod 81: 396-405.

3. Zarelli VE, Ruete MC, Roggero CM, Mayorga LS, Tomes CN (2009) PTP1B Dephosphorylates N-Ethylmaleimide-sensitive Factor and Elicits SNARE Complex Disassembly during Human Sperm Exocytosis. J Biol Chem 284: 10491-10503.

4. Roggero CM, De Blas GA, Dai H, Tomes CN, Rizo J et al. (2007) Complexin/synaptotagmin interplay controls acrosomal exocytosis. J Biol Chem 282: 26335-26343.

5. De Blas GA, Roggero CM, Tomes CN, Mayorga LS (2005) Dynamics of SNARE assembly and disassembly during sperm acrosomal exocytosis. PLoS Biol 3: e323.

6. De Blas G, Michaut M, Trevino CL, Tomes CN, Yunes R et al. (2002) The intraacrosomal calcium pool plays a direct role in acrosomal exocytosis. J Biol Chem 277: 49326-49331.
